# Network Pharmacology Integrated with Transcriptomics Deciphered the Potential Mechanism of *Codonopsis pilosula* against Hepatocellular Carcinoma

**DOI:** 10.1155/2022/1340194

**Published:** 2022-03-27

**Authors:** Zhili Liu, Yuzhe Sun, Hefu Zhen, Chao Nie

**Affiliations:** ^1^College of Life Sciences, University of Chinese Academy of Sciences, Beijing 100049, China; ^2^BGI-Shenzhen, Shenzhen 518083, China; ^3^China National GeneBank, BGI-Shenzhen, Shenzhen 518120, China

## Abstract

Hepatocellular carcinoma (HCC) is the fourth main reason of cancer-related death. *Codonopsis pilosula* is a commonly used traditional Chinese medicine (TCM) for patients with HCC. However, its potential mechanism for treatment of HCC remains unclear. Here, we used transcriptomics and network pharmacology to explore the potential molecular mechanisms of *Codonopsis pilosula*. In our study, twelve differentially expressed genes (DEGs) (5 upregulated and 7 downregulated) of *Codonopsis pilosula* treating HepG2 cells (a kind of HCC cell) were identified. Among the 12 DEGs, HMOX1 may play an essential role. *Codonopsis pilosula* mainly affects the mineral absorption pathway in HCC. We acquired 2957, 1877, and 255 targets from TCMID, SymMap, and TCMSP, respectively. *Codonopsis pilosula* could upregulate HMOX1 via luteolin, capsaicin, and sulforaphane. Our study provided new understanding of the potential pharmacological mechanisms of *Codonopsis pilosula* in treating HCC and pointed out a direction for further experimental research.

## 1. Introduction

There are multiple types of primary liver cancer, of which hepatocellular carcinoma (HCC), the fourth leading cause of cancer-related death overall worldwide, is the most predominant type [1, 2]. During the last few decades, HCC incidence has been increasing at a global level [3, 4], and it is estimated that more than 1 million people will die from HCC in 2030 [5, 6]. In addition to surgical treatments, drugs are the key to HCC therapy [7]. Sorafenib has been the global treatment standard for patients with HCC since 2007 [8], but its efficacy is unsatisfactory [9]. As a widely used alternative therapy, traditional Chinese medicine (TCM) can probably prolong the median survival time and improve the overall survival among patients with HCC [10]. Moreover, some TCMs have been reported to have the ability to assist in elevating the efficacy of sorafenib in the treatment of HCC [11–13].

*Codonopsis pilosula*, a kind of TCM, has anticancer activity and is widely used in adjuvant anticancer therapy [14]. A lot of evidence has shown that many ingredients of *Codonopsis pilosula*, such as Codonopsis pilosula polysaccharide (CPP) and atractylenolide III (ATL), have anti-HCC effects via different pathways. CPP is one of major active constituents in *Codonopsis pilosula*, and it could inhibit the proliferation and motility of HCC cells through the *β*-catenin/TCF4 pathway [15]. CPP1a and CPP1c are two water-soluble homogeneous polysaccharides isolated and purified from *Codonopsis pilosula*, and they could induce HepG2 cell apoptosis by upregulating the ratio of Bax/Bcl-2 and activating caspase-3 [16]. ATL, a sesquiterpenoid extracted from *Codonopsis pilosula*, exerts tumor-suppressive functions in liver cancer via the miR-195-5p/FGFR1 signaling axis [17]. However, *Codonopsis pilosula*, as a kind of Chinese herb, is often used as a whole in clinical practice. There are few reports on the mechanisms of *Codonopsis pilosula* in the treatment of HCC, and its application is greatly limited. The effects of TCM (or herbs of other nations) are not the sum of all active ingredients. In the mixed system of *Codonopsis pilosula*, new effects may emerge that the single active ingredients do not.

In this study, we integrated transcriptomics and network pharmacology to understand the mechanisms of *Codonopsis pilosula* in treating HCC. The differentially expressed genes (DEGs) of *Codonopsis pilosula* were derived from a previous study (GSE115506) [18]. The effective ingredients of *Codonopsis pilosula* and targets were assayed by TCMID, SymMap, and TCMSP [19–21]. The mechanisms of *Codonopsis pilosula* against HCC were assessed by Gene Ontology (GO) and Kyoto Encyclopedia of Genes and Genomes (KEGG) pathway analysis. Furthermore, we found that *Codonopsis pilosula* may regulate mineral absorption through luteolin, capsaicin, and sulforaphane directly targeting HMOX1.

## 2. Materials and Methods

### 2.1. Differentially Expressed Genes Screening

We obtained DEGs of *Codonopsis pilosula* from the GEO database (https://www.ncbi.nlm.nih.gov/geo/) (series: GSE115506; samples: GSM3179695, GSM3179696, GSM3179697, GSM3179698, GSM3179699, and GSM3179700). In GSE115506, total RNA was isolated from HepG2 cells 24 hours after 3 mg/mL *Codonopsis pilosula* aqueous extract treatment in vitro. We performed differential analysis by the Limma R packages [22], and the cutoff value for identifying DEG was set to |log2 fold change| >1 and adjusted *p*value <0.05.

### 2.2. Components and Targets Acquisition

The components and targets of *Codonopsis pilosula* were acquired from TCMID (http://www.megabionet.org/tcmid/) [20], SymMap (http://www.symmap.org/) [21], and TCMSP (https://tcmsp-e.com/) databases [19]. They are all integrative databases of traditional Chinese medicine.

### 2.3. Network Building

We performed the protein-protein interaction (PPI) network analysis using STRING (https://string-db.org/) [23]. The *Codonopsis pilosula*-gene network, protein-protein interaction (PPI) network, and *Codonopsis pilosula*-component-target network were visualized by Cytoscape software [24].

### 2.4. Functional Enrichment Analysis

We conducted Kyoto Encyclopedia of Genes and Genomes (KEGG) pathway analysis and biological process (BP) of Gene Ontology (GO) analysis by R package clusterProfiler [25].

### 2.5. Expression Analysis of HMOX1

The expression of HMOX1 in HCC was obtained through UALCAN, which is a comprehensive and interactive web resource for analyzing cancer OMICS data [26].The expression of HMOX1 after apigenin, luteolin, capsaicin, 4-methylsulfinyl butyl isothiocyanate (sulforaphane), and geniposide treatment was obtained from the GEO database (apigenin series: GSE119552, samples: GSM3377483, GSM3377484, GSM3377485, GSM3377486, GSM3377495, GSM3377496, GSM3377497, and GSM3377498; luteolin series: GSE18740, samples: GSM465440, GSM465441, GSM465442, GSM465443, GSM465444, and GSM465445; capsaicin series: GSE59727, samples: GSM1442972, GSM1442973, GSM1442974, GSM1442975, GSM1442976, GSM1442977, GSM1442978, and GSM1442979; sulforaphane series: GSE28813, samples: GSM713517, GSM713518, GSM713519, GSM713520, GSM713521, GSM713522, GSM713523, and GSM713524; and geniposide series: GSE85871, samples: GSM2286350, GSM2286351, GSM2286248, GSM2286249, GSM2286316, GSM2286317, GSM2286398, and GSM2286399). In GSE18740, mouse BV-2 microglia were treated with 50 *μ*M luteolin for 24 hours; in GSE59727, rat TRPV1-positive neurons were treated with 10 *μ*M capsaicin for 30 minutes; in GSE119552, MCF-7 cells were treated with 10 *μ*M apigenin for 24 hours; in GSE28813, MCF10A cells were treated with 15 *μ*M sulforaphane for 24 hours; and in GSE85871, MCF-7 cells were treated with 10 *μ*M geniposide for 12 hours. We extracted the expression level of the HMOX1 gene from these expression matrices and compared its significance with the *t*-test.

### 2.6. Molecular Docking

The structure of HMOX1 protein was obtained from PDB (https://www.rcsb.org/) [27], and the structures of luteolin, capsaicin, and sulforaphane were acquired from ZINC (https://zinc.docking.org/) [28]. We used AutoDock 4.2 to prepare the PDBQT file and perform molecular docking [29]. Finally, molecular docking maps were visualized through PyMOL.

## 3. Results

### 3.1. *Codonopsis pilosula*-Gene Network and PPI Analysis

**Figure 1:**
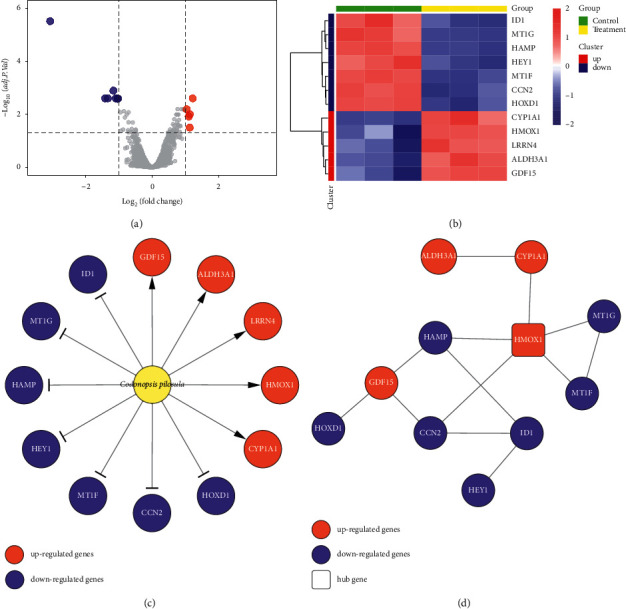
*Codonopsis pilosula*-gene network and PPI analysis. (a) Volcano plot and (b) heatmap of DEGs showed that genes with dramatic changes after *Codonopsis pilosula* treatment. (c) *Codonopsis pilosula*-gene network. (d) The PPI analysis of DEGs.

We identified 12 DEGs (5 upregulated and 7 downregulated) from the GSE115506 data set (Table S1). A volcano plot (Figure 1(a)) and a heatmap (Figure 1(b)) were established to show the distribution of DEGs in HepG2 cells after treating *Codonopsis pilosula*. The cutoff value for identifying DEG was set to |log2 fold change| >1 and the adjusted *p*value <0.05. Accordingly, we built a *Codonopsis pilosula*-gene network (Figure 1(c)). In order to further explore the potential association among these DEGs, we performed a PPI network analysis for the 12 DEGs by STRING [23]. The final PPI network includes 11 nodes and 13 edges (Figure 1(d)). Furthermore, we identified HMOX1, an upregulated gene, as the hub gene because it has the highest degree.

### 3.2. GO and KEGG Analysis

**Figure 2:**
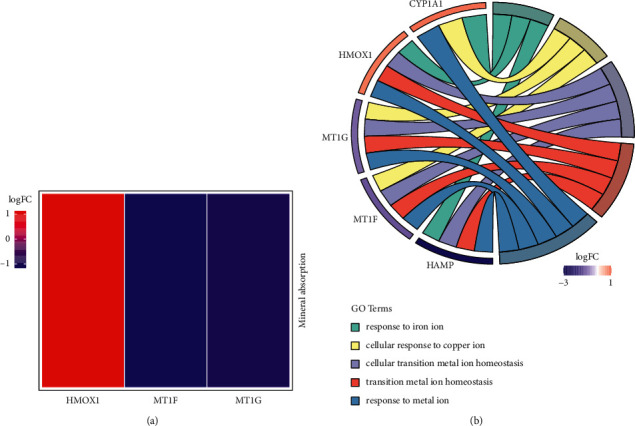
KEGG and GO analysis. (a) KEGG pathway enrichment of *Codonopsis pilosula* treating HepG2 cells. (b) The top 5 biological process in GO terms of *Codonopsis pilosula* treating HepG2 cells.

Through the clusterProfiler R package for KEGG enrichment analysis, we found that only 1 pathway was significantly affected (*p*adjust <0.05) during *Codonopsis pilosula* treatment of HepG2 cells (Figure 2(a)). HMOX1 (hub gene), MT1F, and MT1G were enriched in mineral absorption. In total, 61 biological processes (GO terms) were notably enriched (*p*adjust <0.05) (Table S2). The top 5 biological processes are shown in Figure 2(b). The highly enriched biological processes include responses to iron ions, cellular responses to copper ions, cellular transition metal ion homeostasis, transition metal ion homeostasis, and response to metal ions. These biological processes and pathways are closely related to the metabolism and homeostasis of metal ions, suggesting that *Codonopsis pilosula* mainly affects the mineral absorption pathway in HCC cells.

### 3.3. *Codonopsis pilosula* Reverses HMOX1 Expression in HCC

**Figure 3:**
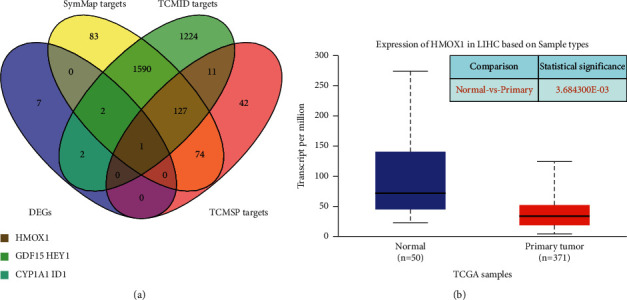
*Codonopsis pilosula* could target HMOX1. (a) Among all targets and 12 DEGs of *Codonopsis pilosula*, HMOX1 is the only common gene. (b) HMOX1 expression is significantly decreased in HCC.

We acquired 2957 targets from TCMID, 1877 targets from SymMap, and 255 targets from TCMSP (Table S3). According to the intersection of these targets and 12 DEGs, we found that HMOX1 is the direct target of *Codonopsis pilosula* in three databases (Figure 3(a)). Our results showed that the expression of HMOX1 was significantly enhanced (Figure 1). Interestingly, HMOX1 was significantly decreased in HCC patients (Figure 3(b)). The abovementioned results suggest that *Codonopsis pilosula* may resist HCC by reversing HMOX1 expression in HCC patients.

### 3.4. *Codonopsis pilosula* Could Upregulate HMOX1 via Luteolin, Capsaicin, and Sulforaphane

**Figure 4:**
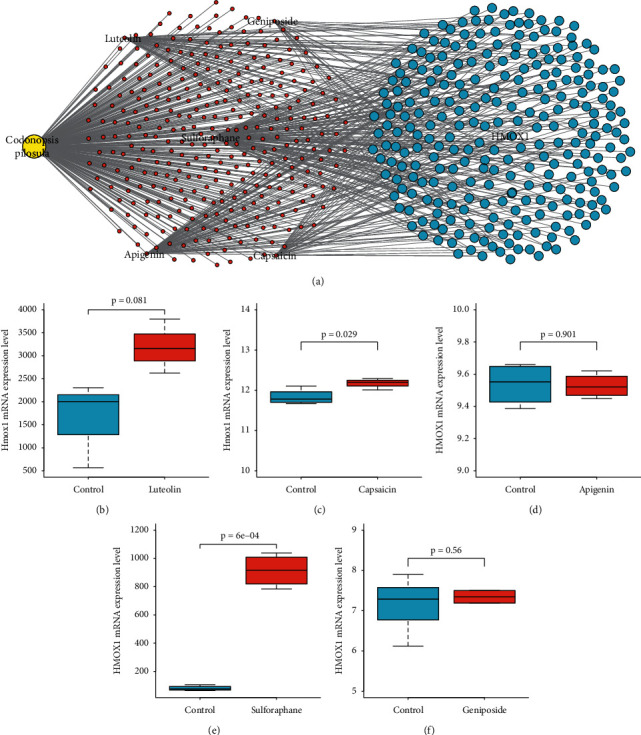
*Codonopsis pilosula* targeted HMOX1 via luteolin, capsaicin, and sulforaphane. (a) *Codonopsis pilosula*-component-target network showed that *Codonopsis pilosula* may target HMOX1 directly through apigenin, luteolin, capsaicin, sulforaphane, and geniposide. (b) Luteolin trends to upregulate Hmox1 in BV-2 cells (*p*=0.081). (c) Capsaicin could significantly upregulate Hmox1 in dorsal root ganglia neurons (*p*=0.029). (d) Apigenin could not affect the expression of HMOX1 in MCF-7 cells (*p*=0.901). (e) Sulforaphane could significantly enhance HMOX1 expression in MCF10A cells (*p*=0.0006). (f) Geniposide has no effect on the expression of HMOX1 in MCF-7 cells (*p*=0.56).

To explore how *Codonopsis pilosula* promotes the expression of HMOX1, we established a *Codonopsis pilosula*-component-target network (Figure 4(a)). The result showed that *Codonopsis pilosula* may directly target HMOX1 through apigenin, luteolin, capsaicin, 4-methylsulfinyl butyl isothiocyanate (sulforaphane), and geniposide. In addition, we detected the expression of HMOX1 after treating with these components (Figures 4(b)–4(f)).  Figure 4(b) showed that luteolin could upregulate Hmox1 in BV-2 cells, although the *p* value is 0.081.  Figure 4(c) showed that capsaicin could significantly promote the expression of Hmox1 in dorsal root ganglia neurons.  Figure 4(d) showed that apigenin could not affect the expression of HMOX1 in MCF-7 cells.  Figure 4(e) manifested that sulforaphane could dramatically enhance the expression of HMOX1 in MCF10A cells.  Figure 4(f) showed that geniposide could not affect the expression of HMOX1 in MCF-7 cells. These results imply that *Codonopsis pilosula* could upregulate HMOX1 in HCC via luteolin, capsaicin, and sulforaphane.

### 3.5. Potential Binding Site between Active Ingredients of *Codonopsis pilosula* and HMOX1 Protein

**Figure 5:**
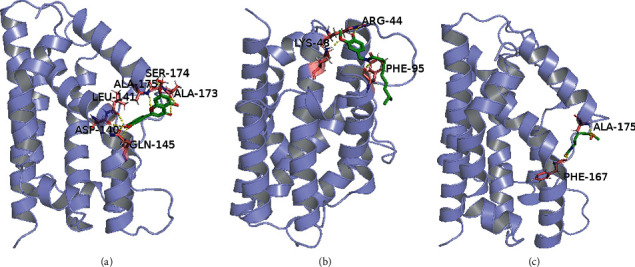
The molecular docking results of luteolin, capsaicin, and sulforaphane. (a) Luteolin may bind to HMOX1 with ASP-140, LEU-141, GLN-145, ALA-173, SER-174, and ALA-175. (b) Capsaicin may combine with HMOX1 by ARG-44, LYS-48, and PHE-95. (c) Sulforaphane may target HMOX1 via PHE-167 and ALA-175.

For exploring potential interaction between active ingredients of *Codonopsis pilosula* (luteolin, capsaicin, and sulforaphane) and HMOX1 protein, we predicted the potential binding site of them via molecular docking. As shown in Figure 5(a), luteolin may directly bind ASP-140, LEU-141, GLN-145, ALA-173, SER-174, and ALA-175 to promote the expression level of HMOX1. Capsaicin may combine with HMOX1 by ARG-44, LYS-48, and PHE-95, thus enhancing the expression level of HMOX1 (Figure 5(b)). Sulforaphane may target HMOX1 via binding PHE-167 and ALA-175, resulting in increased expression of HMOX1 (Figure 5(c)). These results suggest that luteolin, capsaicin, and sulforaphane may promote HMOX1 expression through direct binding.

## 4. Discussion

TCMs are widely used during HCC treatment in China [30]. As with traditional medicine in other nations, herbal medicines are the main form of TCM [31]. Unlike small molecule drugs, herbal medicines contain many components and possess complex targets. Besides, some studies have revealed that miRNAs of herbal medicines may be ingested by the body and regulate the process of disease [32–34]. Complex components and targets limit the exploration of mechanisms in herbal medicines. Although multiple active components of *Codonopsis pilosula* were proved to have anti-HCC potential [15–17], the overall mechanism of *Codonopsis pilosula* is unclear.

In the present study, the *Codonopsis pilosula*-gene network was built by 12 striking DEGs (Figure 1(c)), and we identified HMOX1 as the hub gene via the PPI network (Figure 1(d)). HMOX1 was significantly enriched in the mineral absorption pathway, and the biological processes of its enrichment are primarily related to the metabolism of metal ions (Figures 2(a) and 2(b)). A study reported that there is a remarkable correlation between mineral absorption pathways and HCC development [35]. Furthermore, metal ion metabolism plays an essential role in the progression and treatment of HCC [36, 37]. Consequently, *Codonopsis pilosula* is highly likely to treat HCC via targeting HMOX1 to affect the mineral absorption pathway.

HMOX1 (heme oxygenase-1) is a stress-induced enzyme that catalyzes the degradation of heme to carbon monoxide, iron, and biliverdin [38]. The byproducts of HMOX1 enzymatic activity are cytoprotective because of their antioxidant and anti-inflammatory properties, showing that HMOX1 is a potential therapeutic target in many diseases [39]. Our results revealed that HMOX1 was significantly decreased in HCC patients (Figure 3(b)), and *Codonopsis pilosula* could distinctly enhance the expression of HMOX1 in HepG2 cells (Figure 1), suggesting that *Codonopsis pilosula* may reverse the expression pattern of HMOX1 in the HCC environment. Interestingly, HMOX1 overexpression could inhibit the growth, migration, and invasion in vivo, as well as higher HMOX1 expression was also associated with favorable disease-free survival of HBV-HCC patients who underwent hepatectomy [40]. These results indicate that *Codonopsis pilosula* is likely to improve the survival of HCC patients by promoting the expression of HMOX1, and is a potential adjuvant therapy for HCC.

Through the network pharmacology strategies, we built a *Codonopsis pilosula*-component-target network (Figure 4(a)). The network showed that *Codonopsis pilosula* may directly target HMOX1 via apigenin, luteolin, capsaicin, sulforaphane, and geniposide. To verify this result, we examined the effect of luteolin on Hmox1 expression in mouse BV-2 microglia (GSE18740), capsaicin on Hmox1 expression in rat TRPV1-positive neurons (GSE59727), apigenin on HMOX1 expression in human breast cancer cells MCF-7 (GSE119552), sulforaphane on HMOX1 expression in human breast epithelial cells MCF10A (GSE28813), and geniposide on HMOX1 expression in human breast cancer cells MCF-7 (GSE85871) (Figures 4(b)–4(f)). The results indicated that luteolin, capsaicin, and sulforaphane could increase the expression of HMOX1 expression in vitro, although not in HCC cells. Luteolin, a natural flavonoid, plays multiple roles in the anti-HCC process. Growth inhibition of luteolin on HCC cells is induced via multiple signaling pathways of TGF-*β*1 pathways, p53 pathways, Fas/Fas-ligand pathways [41], ER stress [42], and AKT/OPN pathway [43]. Besides, a recent study reported that luteolin could significantly inhibit HCC growth and cause apoptosis and cell cycle arrest in vitro and significantly suppress HCC growth in vivo via upregulating miR-6809-5p [44]. Capsaicin is a natural vanilloid and may inhibit the growth of SK-Hep-1 hepatocellular carcinoma cells by inducing apoptosis via Bcl-2 downregulation and caspase-3 activation [45]. Moreover, capsaicin could induce apoptosis in HepG2 cells by reducing the levels of xIAP and cIAP1 proteins, which are inhibitors of caspase-3 activation [46]. Interestingly, both luteolin and capsaicin are able to assist sorafenib to produce better anti-HCC therapeutic effects [47, 48]. Sulforaphane, a member of the isothiocyanate family, has exhibited promising inhibitory effects on breast cancer, lung cancer, liver cancer, and other malignant tumors [49]. Some studies revealed that sulforaphane could induce apoptosis [50] and enhance the radiation sensitivity [51] in HCC.

**Figure 6:**
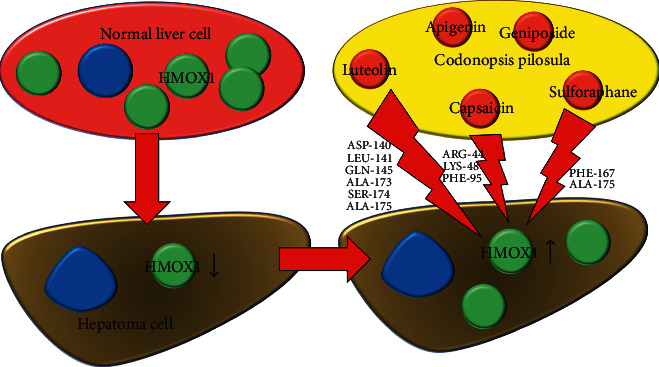
Schematic diagram of *Codonopsis pilosula* for HCC treatment.

Notably, there are about 200 phytometabolites in *Codonopsis pilosula*, and the main bioactive ingredients include polysaccharides, polyyne and polyacetylene glycosides, lignans, penoids, alkaloids, flavonoids, and lactones [52]. Polysaccharides are large-molecule components in *Codonopsis pilosula*, which have a significant inhibitory effect on gastric cancer and lung cancer, in addition to liver cancer [53]. Although luteolin, capsaicin, and sulforaphane are not the most abundant ingredients of *Codonopsis pilosula*, they are essential for understanding the pharmacological effects of *Codonopsis pilosula*. Tang et al. found that *Codonopsis pilosula* may play an antigastric cancer role through luteolin [54], suggesting that luteolin may play an important role in the anticancer effects of *Codonopsis pilosula*. Furthermore, several studies showed that luteolin [55], capsaicin [56], and sulforaphane [57] could target HMOX1 and significantly enhance its expression level. Luteolin, capsaicin, and sulforaphane are components of *Codonopsis pilosula*, but their quantitative studies in *Codonopsis pilosula* are insufficient. It is reported that the content of luteolin in *Codonopsis thalictrifolis* is 0.7% via HPLC [58]. It is important to notice that *Codonopsis thalictrifolis* is not *Codonopsis pilosula*, although they belong to the *Codonopsis* genus, and there may be great differences in chemical composition between them. Nonetheless, as a reference, the data implied that the content of the luteolin in *Codonopsis pilosula* may be less than 0.1% or even 0.01%. The lowest dose of luteolin that has been reported to produce anti-HCC effects in rats is 0.2 mg/kg via intraperitoneal injection [59]. In addition, orally administered luteolin (0.2 mg/kg) could produce anticolon cancer effects in rats [60]. Fuzheng Jiedu Xiaoji formulation (including 15 g of *Codonopsis pilosula*) could inhibit HCC progression in patients [61]. Jian Pi Li Qi Decoction (including 20 g of *Codonopsis pilosula*) could improve the prognosis of patients with HCC [62]. Therefore, it is likely that the effective concentration of luteolin can be reached in the application of *Codonopsis pilosula*. At present, there are no quantitative studies on capsaicin and sulforaphane in *Codonopsis pilosula*. Studies showed that capsaicin (2 mg/kg) [63] and sulforaphane (50 mg/kg) [64] could inhibit the growth of HCC in xenograft mice. Compared with the effective dosage of capsaicin and sulforaphane, the application of *Codonopsis pilosula* (15–20 g) is higher. Consequently, luteolin, capsaicin, and sulforaphane are likely to reach effective concentrations in the clinical application of *Codonopsis pilosula*. These studies suggest that *Codonopsis pilosula* is most likely to exert anti-HCC effects via luteolin, capsaicin, and sulforaphane (Figure 6). In fact, although our study showed that luteolin, capsaicin, and sulforaphane may play roles in the adjuvant treatment of HCC by *Codonopsis pilosula*, they may not be the main active ingredients of *Codonopsis pilosula*. As an herbal medicine, *Codonopsis pilosula* contains a variety of ingredients. An inulin fructan from *Codonopsis pilosula* possessed potential anti-HCC effects (inhibiting proliferation and inducing apoptosis) on Huh-7 and HepG2 cells without side effects on normal cells [65]. In addition, a novel fructose-enriched polysaccharide from *Codonopsis pilosula* inhibited HepG2 cell proliferation and promoted apoptosis [66]. Taken together, apoptosis may be one of the anti-HCC pathways of *Codonopsis pilosula*.

## 5. Conclusions

The present study explored the effects of *Codonopsis pilosula* in the treatment of HCC via transcriptomics and network pharmacology. We revealed the transcriptome changes of HCC cells induced by *Codonopsis pilosula*. In addition, *Codonopsis pilosula* is likely to upregulate HMOX1 directly through luteolin, capsaicin, and sulforaphane, thus affecting the mineral absorption pathway in HCC cells. This study provides clues to comprehend the potential mechanisms of *Codonopsis pilosula* in treating HCC. Of course, these conclusions require further experimental support.

## Data Availability

The data used to support the findings of this study are available from the corresponding author upon request.

## Supplementary Materials

Supplementary Materials Tables S1–S3 in the Supplemental files. Table S1: twelve DEGs in HepG2 cells after *Codonopsis pilosula* treatment. Table S2: all significantly enriched biological processes after *Codonopsis pilosula* treatment. Table S3: targets of *Codonopsis pilosula*.
