# Supplementary material for: Network Pharmacology Integrated with Transcriptomics Deciphered the Potential Mechanism of Codonopsis pilosula against Hepatocellular Carcinoma
Source: Evid Based Complement Alternat Med. 2022 Mar 27;2022:1340194. doi: 10.1155/2022/1340194 (PMC8977304; doi:10.1155/2022/1340194)
Supplement: Supplementary Materials — Tables S1–S3 in the Supplemental files. Table S1: twelve DEGs in HepG2 cells after Codonopsis pilosula treatment. Table S2: all significantly enriched biological processes after Codonopsis pilosula treatment. Table S3: targets of Codonopsis pilosula. [file 1340194.f1.pdf]

Supplemental Files

Table S1 12 DEGs in HepG2 cells after *Codonopsis pilosula* treatment

gene	logFC	AveExpr	P.Value	adj.P.Val
HAMP	-3.06362	8.044501	1.49E-10	3.10E-06
ID1	-1.41348	10.70832	6.70E-07	0.002504
HEY1	-1.33231	7.714727	5.38E-07	0.002504
MT1F	-1.16552	10.63498	1.23E-07	0.001274
HOXD1	-1.09257	9.57921	6.33E-07	0.002504
CCN2	-1.03513	9.426633	1.01E-06	0.002628
MT1G	-1.03233	12.25778	8.02E-07	0.002504
ALDH3A1	1.035084	7.035056	4.05E-06	0.006465
LRRN4	1.097444	8.685654	1.35E-05	0.012181
HMOX1	1.124853	8.500588	5.56E-05	0.031773
GDF15	1.128426	10.34754	9.23E-06	0.0101
CYP1A1	1.213903	6.108217	8.45E-07	0.002504

Table S2 All significantly enriched biological processes

ID	Description	p.adjust	geneID
GO:0010039	response to iron ion	0.000155	HAMP/HMOX1/CYP1A1
GO:0071280	cellular response to copper ion	0.000155	MT1F/MT1G/CYP1A1
GO:0046916	cellular transition metal ion homeostasis	0.000155	HAMP/MT1F/MT1G/HMOX1
GO:0055076	transition metal ion homeostasis	0.000183	HAMP/MT1F/MT1G/HMOX1
GO:0010038	response to metal ion	0.000183	HAMP/MT1F/MT1G/HMOX1/CYP1A1
GO:0071276	cellular response to cadmium ion	0.000183	MT1F/MT1G/HMOX1
GO:0046688	response to copper ion	0.000213	MT1F/MT1G/CYP1A1
GO:0007584	response to nutrient	0.000222	HAMP/ALDH3A1/HMOX1/CYP1A1
GO:0043392	negative regulation of DNA binding	0.000304	ID1/HEY1/HMOX1
GO:0071248	cellular response to metal ion	0.000304	MT1F/MT1G/HMOX1/CYP1A1
GO:0031667	response to nutrient levels	0.000315	HAMP/ALDH3A1/HMOX1/GDF15/CYP1A1
GO:0010043	response to zinc ion	0.000315	HAMP/MT1F/MT1G
GO:0009991	response to extracellular stimulus	0.000332	HAMP/ALDH3A1/HMOX1/GDF15/CYP1A1
GO:0046686	response to cadmium ion	0.000332	MT1F/MT1G/HMOX1
GO:0071241	cellular response to inorganic substance	0.000344	MT1F/MT1G/HMOX1/CYP1A1
GO:0007568	aging	0.001238	HAMP/CCN2/ALDH3A1/CYP1A1
GO:0010273	detoxification of copper ion	0.001444	MT1F/MT1G
GO:1990169	stress response to copper ion	0.001444	MT1F/MT1G
GO:0061687	detoxification of inorganic compound	0.001667	MT1F/MT1G
GO:0051101	regulation of DNA binding	0.001667	ID1/HEY1/HMOX1
GO:0033189	response to vitamin A	0.001667	HAMP/CYP1A1
GO:0097501	stress response to metal ion	0.001667	MT1F/MT1G
GO:0036293	response to decreased oxygen levels	0.001667	CCN2/ALDH3A1/HMOX1/CYP1A1
GO:0070482	response to oxygen levels	0.002074	CCN2/ALDH3A1/HMOX1/CYP1A1
GO:0001889	liver development	0.002153	HAMP/HMOX1/CYP1A1
GO:0061008	hepaticobiliary system development	0.00216	HAMP/HMOX1/CYP1A1
GO:0071294	cellular response to zinc ion	0.002522	MT1F/MT1G
GO:0031960	response to corticosteroid	0.002561	HEY1/CCN2/ALDH3A1
GO:0051100	negative regulation of binding	0.002825	ID1/HEY1/HMOX1
GO:0046685	response to arsenic-containing substance	0.003569	HMOX1/CYP1A1
GO:0097421	liver regeneration	0.003691	HAMP/HMOX1
GO:0006882	cellular zinc ion homeostasis	0.005392	MT1F/MT1G
GO:0006778	porphyrin-containing compound metabolic process	0.005627	HMOX1/CYP1A1
GO:0055069	zinc ion homeostasis	0.005627	MT1F/MT1G
GO:0009636	response to toxic substance	0.007775	MT1F/MT1G/CYP1A1
GO:0045926	negative regulation of growth	0.008127	MT1F/MT1G/GDF15
GO:0032233	positive regulation of actin filament bundle assembly	0.012111	ID1/CCN2
GO:0033013	tetrapyrrole metabolic process	0.012111	HMOX1/CYP1A1
GO:0006879	cellular iron ion homeostasis	0.015028	HAMP/HMOX1
GO:0031100	animal organ regeneration	0.015928	HAMP/HMOX1

GO:0048545	response to steroid hormone	0.016967	HEY1/CCN2/ALDH3A1
GO:0001666	response to hypoxia	0.019308	ALDH3A1/HMOX1/CYP1A1
GO:0051098	regulation of binding	0.019711	ID1/HEY1/HMOX1
GO:0055072	iron ion homeostasis	0.019711	HAMP/HMOX1
GO:0042493	response to drug	0.019711	ALDH3A1/HMOX1/CYP1A1
GO:0042692	muscle cell differentiation	0.01975	HAMP/HEY1/GDF15
GO:0033273	response to vitamin	0.020086	HAMP/CYP1A1
GO:0032231	regulation of actin filament bundle assembly	0.025751	ID1/CCN2
GO:0048732	gland development	0.027059	HAMP/HMOX1/CYP1A1
GO:0043500	muscle adaptation	0.032561	HAMP/HMOX1
GO:0045446	endothelial cell differentiation	0.032561	ID1/HEY1
GO:0006805	xenobiotic metabolic process	0.032561	ALDH3A1/CYP1A1
GO:0003012	muscle system process	0.032561	HAMP/CCN2/HMOX1
GO:0071466	cellular response to xenobiotic stimulus	0.03416	ALDH3A1/CYP1A1
GO:0009410	response to xenobiotic stimulus	0.036772	ALDH3A1/CYP1A1
GO:0051384	response to glucocorticoid	0.038309	HEY1/ALDH3A1
GO:0003158	endothelium development	0.038616	ID1/HEY1
GO:0098754	detoxification	0.038616	MT1F/MT1G
GO:0002262	myeloid cell homeostasis	0.041821	HAMP/HMOX1
GO:0051017	actin filament bundle assembly	0.046259	ID1/CCN2
GO:0061572	actin filament bundle organization	0.047835	ID1/CCN2

Table S3 Targets of *Codonopsis pilosula*

Targets from TCMID			Targets from SymMap			Targets from TCMSP
SRPX2	DCAF16	CRP	A2M	HRAS	TOP2A	PGR
BIRC2	C7	STAT5B	A2ML1	HSD3B2	TP53	NCOA2
DDB1	ACTR1A	ZNF697	AAGAB	HSPA5	TPI1	NR3C2
ZNF562	CTCFL	CENPE	ABAT	HTR2A	TRIP11	CHRM3
ZNF682	HSPA9	PROX1	ABCA1	HYAL1	TRPV1	F2
ZNF230	ZNF700	ZNF189	ABCB1	ICAM1	TRPV3	CHRM1
ZNF527	SLC38A8	ZNF561	ABCB11	IFNG	TRPV4	AR
CHRNA2	SLC38A7	ANKRD28	ABCC2	IGF1	TSHR	NOS3
TBK1	DEFB129	GFI1	ABL1	IGF1R	TWIST1	RXRA
ZNF584	ZNF215	MYBL2	ACACA	IGHG1	TYR	HTR2A
ZNF438	ASPG	CHRNA9	ACE	IGHMBP2	UNC119	CHRM2
ZNF831	GZF1	ACTB	ACHE	IGSF3	USH2A	ADRB2
ZNF141	TMEM41B	ZNF778	ACOX1	IKBKG	VCAM1	SLC6A4
ZNF254	ZNF768	ZNF280D	ACTA1	IKZF1	VCAN	ADRB1
COL23A1	ZNF440	ZNF519	ACTA2	IL10	VEGFA	PTGS2
DYNC1LI2	CHGA	CBL	ACTB	IL12B	VIM	PDE3A
VCAM1	ACTR8	ZNF747	ACTC1	IL13	WNT5A	ADRA1A
SELE	ZNF569	CYP1A1	ACTG1	IL1A	XDH	ADRA1B
GJA1	PLA2G6	ZNF37A	ACTG2	IL1B	XIAP	SLC6A3
CHRNA7	HTR3E	AEBP2	ADAMTS13	IL2	ZBTB42	GABRA1
ASB6	GSDMA	COL9A2	ADCY2	IL21	ZFP57	GABRA2
ZNF442	ZNF532	ZNF33A	ADGRE2	IL21R	ZFPM2	camC
KLRG2	ZNF568	ZIM3	ADGRG2	IL2RA	ZIC1	cobT
ZBTB16	ZNF80	ZKSCAN3	ADGRG6	IL2RB	ZIC3	PTGS1
ZNF98	NTSR1	ZNF43	ADRA1A	IL2RG	ZNF335	E
PFN1	CASKIN1	ACTRT1	ADRA2A	IL4	ZNF408	SLC6A2
TNKS2	ZNF18	ZBTB9	ADRB1	IL6	ZNF41	IGHG1
ZNF142	FGF2	ZNF280A	ADRB2	INS	ZNF423	TRPV1
ASB18	ZNF835	ZNF143	AFP	INSR	ZNF469	GABRA6
ANKRD61	ZNF223	NFKB2	AGA	INVS	ZNF513	BCHE
UCP1	TANC2	ZFP69B	AKR1B1	IRAK4	ZNF592	PLA2G1B
FAS	PRDM16	SPTLC3	AKR1C3	ISYNA1	ZNF644	P23008
CA8	HTR2A	HIC1	AKT1	ITGA2B	ZNF687	fhuA
ZNF713	ASB4	GABBR1	ALB	ITGAL	ZNF711	fabB
CA9	MYCN	ZNF304	ALOX5	ITGB1	ZNF81	NCOA1
N/A	NTRK2	ZNF107	ALPL	JAK1	CCK	LCAT
CCNG2	GPX5	ZNF181	AMHR2	JAK2	CCNB1	RHO
ZNF589	TAGLN2	AATK	ANK1	JUN	CDC25C	GABRA5
ANKRD9	PLAUR	EGR1	ANK2	KANK1	BAK1	GABRA3
ZNF33B	SP6	ZNF222	ANK3	KCNH2	BARD1	GOT1

ZNF431	SOD1	PRG2	ANKRD1	KDR	BAX	GRIA1
EMP1	FYN	ZNF426	ANKRD26	KIRREL3	BCL3	AKR1B1
FOXP3	KLF10	ATF3	ANKRD55	KLF1	GADD45A	CTSD
ZNF17	OSR1	ZNF345	ANKS6	KLF6	BID	aspC
ZNF212	ZNF14	ZNF550	APAF1	KLK3	DFFA	nprS
COL4A4	ZNF454	ZNF99	APC	KLK4	CNN3	TPI1
ZNF343	ZNF738	EGR3	APP	KLKB1	ALPI	ME2
ZNF337	ZNF57	IDH3B	AR	KMT2A	E2F1	NOS1
HDAC7	NOS2	NPY	ASB10	KRT6B	ANGPT1	ALOX5
ZFAT	ZNF267	RAET1E	ASCL1	KRT85	BIRC2	ABAT
ZSCAN32	UGT2B10	ZNF268	ATM	LAMA1	CASP9	PYGM
HABP2	SFTPA2	ACTR10	ATP5B	LAMA2	RUNX1T1	yrC
IFI44	IL9	MYC	BAD	LAMA3	SMAD7	purA
ANKRD36	SRC	ZNF692	BAG3	LAMB1	GZMB	metK
ANKRD30B	ACTL7B	COL4A1	BCHE	LAMB3	GABPB1	GIG18
HINT1	ZNF559	ZNF415	BCL2	LCAT	GAP43	Q70AC7
ZNF620	ZBTB42	VEGFA	BCL2L1	LCK	GCG	GOT2
ASGR1	HPCAL4	EPHB3	BGLAP	LDLR	JUNB	GRIA2
COL5A1	KANK3	ZNF630	BIRC5	LEP	F2RL1	GRIK2
ZNF320	HSPA12B	CCNA2	BRCA1	LGALS1	FABP4	PTPN1
ZNF771	CYP2C9	ZFPM1	BTK	LITAF	FABP1	PRSS3
PARK2	PPP1R16A	ZFP2	C1S	LMAN2L	FABP5	ISYNA1
ZNF239	PROM2	ZBTB43	C2	LPL	HP	PRKACA
CYP11A1	FEM1B	ZNF461	C4A	LYZ	SFN	MAOB
UGT2B11	EGR2	ZNF687	C4B	MAFB	FOSB	RELA
ZNF605	ZNF195	SCGB2B2	C6	MAN2B1	IGFBP1	AKT1
HSPA13	SCD	ZNF485	C7	MAOB	GYPA	BCL2
ANK2	ZBTB21	ZNF606	C8A	MAP2	NFE2L2	FOS
BTK	ZNF705B	STXBP3	C8B	MAP2K1	PTPRG	MAPK1
ZNF674	GPX6	TPR	CA12	MAP3K7	POU2F1	CASP3
ZNF536	ZSCAN2	SVEP1	CA2	MAPK1	RBP2	CHUK
KLF4	CHRM3	STAC	CA4	MAPK3	OPCML	MMP1
CYP2A7	MMP1	VWF	CA5A	MAPK9	KLK10	JUNB
IL10	COL20A1	FSCN1	CA8	MASP1	PSMD3	HMOX1
DEDD	COL14A1	ZNF662	CACNA1H	MASP2	TRPM2	CREB1
CD72	CA14	ZNF486	CALCA	MCL1	KLF7	GAP43
ZNF441	ZNF660	ZSCAN1	CALM3	MDM2	TNFSF10	ABCB1
ZNF648	ZNF232	ACTR3	CASP3	MECOM	TAC1	NFE2L2
ZNF699	FMO1	HACE1	CASP7	MEFV	UCP1	GFAP
ZNF624	KLF13	CHRNA10	CASP8	MET	SLC7A5	F2RL1
BTBD11	ZBTB48	ZNF567	CASQ1	MGAT2	SLC2A4	GATA1
PTPRA	ADRB1	ZNF880	CASQ2	MIB1	TEP1	GLUL
CXCL2	ITPR1	CNTN1	CATSPER1	MLPH	SLPI	TGFA
SLC35A1	DNM1L	TWIST1	CBL	MMP1	KLF10	KLK3

CD4	CLEC19A	CYP2A6	CBS	MMP13	TIMP1	CTRC
MAZ	ZIC1	ZNF48	CCNA2	MMP19	TIMP4	HSD3B2
KDM6A	ZNF837	CRELD2	CCND1	MMP2	TRIM26	RAC1
ZFP92	CHAT	ANKRD49	CCND2	MMP20	SP1	POMC
STC2	PLG	SHANK2	CCR5	MMP21	SPI1	UCP1
IKZF5	NOTCH4	ANKRD44	CD4	MMP3	MADD	CACNA1H
PRRC2C	TF	EPO	CD40	MMP7	AHSA1	PGP
ZSCAN29	VIM	BCL3	CD40LG	MMP8	PSME3	HBB
CLEC2D	ZNF444	ZKSCAN2	CD44	MMP9	TNFRSF10A	GYPA
ACTL7A	VEZF1	ZNF774	CD59	MR1	CFLAR	SPI1
ZNF790	ZNF534	ZSCAN4	CD80	MS4A2	VNN1	TAC1
CCS	ZNF786	EGR4	CD86	MSMO1	EEF1E1	TACR1
GPNMB	ANKRD16	ZNF91	CDK2	MST1	PTGES	DRD1
CHRNB4	GABBR2	ANKRD65	CDK4	MTRR	INMT	SCN5A
COL25A1	ZNF587B	NEFM	CDK6	MYC	ARC	NQO2
ZNF773	ZNF639	TSHR	CDKN1A	NCOA1	SPDEF	ADRA2C
FLRT2	PRDM13	CCDC159	CDKN1B	NDUFS4	HEY1	ampC
NEUROD1	COL6A3	AKT1	CDKN2A	NECTIN1	NOX3	LTA4H
ZNF627	ACSL6	CLEC4A	CDKN2B	NEUROD1	ALG5	PKIA
ANKRD66	ANKRD50	KLRD1	CDKN2C	NFATC1	HERC5	ADRA2A
ZNF286A	CLEC4M	ZNF570	CDKN2D	NFKB1	MOGAT2	ADRA2B
ITGB1	ANKRD5	CDKN1B	CES1	NFKB2	VTCN1	ADRA1D
ZBTB24	ANKRD22	LCK	CFB	NFKBIA	NUF2	linB
NOS1	ZNF248	ZNF544	CFD	NGF	TMPRSS13	PLA2G2E
ORC2	PPP1R16B	ZNF729	CFH	NIN	PLB1	tesA
IFI27	SPP1	ADTRP	CFI	NOS1	ENPP7	CXCL8
CLEC2A	ZNF736	ZKSCAN4	CHAT	NOS2	PGP	PPARA
ZNF334	ZNF777	ZNF805	CHEK2	NOS3	DHFRP1	EP300
EVPLL	KLF1	SLC38A11	CHKB	NOTCH1	GUSBP1	FABP1
ZNF749	MAPK3	IFI6	CHRM1	NOTCH2	CDC2	RBP2
AVP	FGF4	ZNF829	CHRM3	NOTCH3	PVRL1	IL6
POTEJ	IL3RA	ARHGEF7	CHRNA1	NPC1	DHPS	DUOX2
ZFY	NFKBIB	PON2	CHRNA4	NPEPPS	FCGR2A	CD80
CLECL1	LITAF	RARG	CHRNA6	NQO1	AATK	CD86
ZSCAN23	TTR	SLC38A9	CHRNA7	NQO2	ABTB1	CD40
ANKRD20A4	YES1	ZNF34	CHRNA9	NR1H4	ACR	PLAU
ESPN	FMO4	ZNF695	CHRNB1	NR3C1	ACSL3	MAOA
ZNF35	COL4A5	CLEC1A	CHRNB2	NR3C2	ACTBL2	CTRB1
GTF3A	CLCC1	SP8	CHRND	NRL	ACTL10	CHRNA7
ZNF323	DTYMK	PAX8	CHRNE	NSD1	ACTL6A	F10
ZNF879	S100A2	ZNF335	CHRNG	NSD2	ACTL6B	TOP2A
ZBTB37	ZFP41	ZNF707	CLCN1	NTNG1	ACTL7A	CALM1
TOPBP1	ZNF417	APOH	CLCN2	NTRK1	ACTL7B	KCNH2
IKZF2	ZIC3	NFKBIE	CLCN4	OCLN	ACTL8	F7

ZNF770	ANKS1A	CDH1	CLCN7	ODC1	ACTL9	KCNMA1
INMT	ZNF84	ZBTB3	CLCNKA	OPRD1	ACTR10	HSP90AA1
CD46	IL6	ANKRD60	CLMP	OPRK1	ACTR1A	NOS2
PRDM10	MYNN	CD276	CMA1	OPRM1	ACTR1B	ESR1
ZNF709	ZNF165	NFKBIZ	CNTN1	P2RX1	ACTR2	PPARG
RBAK	MAFB	S100A10	COL10A1	PAX8	ACTR3	ESR2
SLC38A1	ZKSCAN5	LIG4	COL11A1	PCNA	ACTR3B	MAPK14
ZNF772	ZNF19	SLC19A1	COL14A1	PCYT1A	ACTR3C	GSK3B
ZNF286B	SLC1A1	COL6A1	COL17A1	PDGFRB	ACTR8	CDK2
ZNF560	KIDINS220	TUBA4A	COL18A1	PDHX	ACTRT1	PIK3CG
ZNF71	HLA-F	SOWAHC	COL1A1	PER2	ACTRT2	CHEK1
HSP90AA1	COLQ	ZNF566	COL25A1	PGR	ACTRT3	PRSS1
ZBTB11	ZNF138	OSR2	COL27A1	PHF21A	ADAM19	PIM1
ZNF621	REG3A	CRABP2	COL3A1	PIGN	ADGRD1	CCNA2
MLL3	PRDM8	PPP2R3A	COL5A2	PLA2G6	ADGRD2	TEP1
SERPINE1	HOXB9	ZNF784	COL9A1	PLAG1	ADGRE1	IL1B
CLCN3	CHRND	ZNF850	COL9A2	PLAT	ADGRE3	PTGER3
ANKRD52	BGLAP	CYP2F1	COL9A3	PLAU	ADGRF1	NPEPPS
GABPB2	ZNF69	OLR1	COLQ	PLAUR	ADGRF2	IGF1
WIZ	ZNF843	SP2	CORIN	PLG	ADGRF3	SPDEF
ZNF227	SLC7A11	PTPRZ1	CPT1A	PLK1	ADGRF4	TERT
SYNPO	HSPA14	ZNF462	CRAT	POGZ	ADGRF5	TIMP3
PRDM4	APEH	CHRNB3	CREB1	POLD1	ADGRG3	ACHE
HGF	ZNF675	CHRNE	CSF2	POMC	ADGRG4	DPP4
ZNF814	ASB14	CXCR2	CSF2RB	PON1	ADGRG5	CHRM5
ZFX	ABCA1	ZNF791	CTCF	PON2	ADGRG7	OPRM1
POSTN	ZNF667	ZNF781	CTNNB1	PON3	ADGRL1	APOB
MTF1	ZFP30	CYP2S1	CTRC	POR	ADGRL2	LPL
EEF2	ZNF878	BTF3	CTSD	POU2AF1	ADGRL3	ATP5B
COL1A2	ZNF420	CNR1	CTSG	PPARA	ADGRL4	HP
ANGPT4	AGT	ASB5	CTSH	PPARD	ADRA1D	PDHX
ZNF559-ZNF177	BCL6	HTR3C	CUBN	PPARG	ADTRP	MOGAT2
MED28	ZNF502	NRL	CXCL8	PRDM16	AEBP2	PPARGC1A
ZNF710	IL8	ZNF521	CXCR4	PRDM8	ALDH1A1	TNF
FGF6	ZNF557	FLT3	CYCS	PRG4	AMIGO1	CHRM4
ZNF684	SH3KBP1	ZIC2	CYP11A1	PRKACA	AMIGO2	HTR2C
BARD1	FANK1	ZNF45	CYP19A1	PRKCA	ANGPT2	EGFR
ZNF407	MUC4	NACC2	CYP1A2	PRKCD	ANGPT4	CCND1
BCL11A	ANKRD45	SNAI2	CYP2C19	PRKCZ	ANKDD1B	BCL2L1
ZNF563	IRS1	GLIS3	CYP2C9	PRKDC	ANKEF1	CDKN1A
PLAU	COL7A1	ZNF782	CYP2D6	PRKN	ANKFY1	CASP9
ZNF354C	ZNF154	PRKAR2B	CYP2E1	PRSS1	ANKHD1	MMP2
BCL2L1	PTGS2	SP4	DCC	PRSS12	ANKK1	MMP9

HIF1A	ZNF615	FEZF1	DDX3X	PRTN3	ANKRA2	IL10
JHDM1D	NGF	ZNF510	DHFR	PTDSS1	ANKRD10	RB1
PANK3	GNG10	ADRB2	DNMT1	PTEN	ANKRD16	CDK4
KANK1	CREB1	ZNF780A	DRD1	PTGER3	ANKRD17	JUN
ZNF549	CAMP	ZNF221	DUOX2	PTGS1	ANKRD18A	TP53
CEBPB	NOTCH1	MAPK1	EDN1	PTGS2	ANKRD18B	XDH
ZNF783	ZBTB32	ASB10	EDNRA	RAC1	ANKRD2	topA
LCE1B	ZNF780B	NFKB1	EEF2	RAF1	ANKRD20A2P	MDM2
ZNF480	ZNF302	ZNF641	EGF	RB1	ANKRD20A4P	APP
ANKRD6	ZNF737	TUBB	EGFR	RELA	ANKRD22	PCNA
POGZ	KLRK1	COL2A1	EHMT1	RELN	ANKRD23	ERBB2
FEZ1	GM2A	ZNF585A	EIF6	REN	ANKRD24	CASP7
ACTR3C	KLF15	ZNF418	ELANE	REST	ANKRD27	ICAM1
COL16A1	KRBOX1	KLRB1	ENOX2	RFXANK	ANKRD28	MCL1
WT1	FMO3	ZNF202	ENPP1	RFXAP	ANKRD29	BIRC5
ANKK1	ZNF253	POTEH	EP300	RHO	ANKRD30A	IL2
RFXAP	ZNF324B	ZNF728	EPOR	RIPK4	ANKRD30B	CCNB1
ZNF213	HSPA2	EZR	ERBB2	RNASEL	ANKRD30BL	TYR
TRPA1	SLC6A3	IGSF3	ERBB4	RNF135	ANKRD35	IFNG
CYP2E1	ZKSCAN7	CTTNBP2	ESPN	RPS6KA3	ANKRD36	IL4
CXCR3	HSPH1	STAR	ESR1	RREB1	ANKRD36C	XIAP
CYP4F3	KLF6	C4BPB	ESR2	RUNX2	ANKRD37	SLC2A4
ZNF90	ZNF554	CCR6	EZH2	RXRA	ANKRD39	INSR
ZNF740	ZNF679	ZNF575	F10	RXRB	ANKRD42	CD40LG
ZNF124	ZBTB7A	ZNF547	F11	SCN5A	ANKRD44	PTGES
CYP2D6	PRDM5	BIRC5	F12	SCP2	ANKRD46	NUF2
CYP2B6	SP5	PRDM6	F2	SELE	ANKRD49	ADCY2
COL4A3	IFI44L	COL3A1	F7	SEMA3D	ANKRD50	MET
COL4A6	NFKBIA	EIF2AK2	F9	SEMA3E	ANKRD52	CNN3
ZFP28	ZNF157	PRDM9	FAM20A	SEMA4A	ANKRD53	PTEN
SLC6A4	RIPK4	MR1	FAM20C	SEMA5A	ANKRD54	GUSBP1
ZNF430	ZSCAN5C	LTB	FAS	SERPINE1	ANKRD6	SLC22A5
MTIF2	LDLR	ZNF396	FCER2	SETBP1	ANKRD60	PCYT1A
ZSCAN16	FEM1A	ZSCAN9	FCGR3A	SIRT1	ANKRD61	CA2
CLEC7A	TXNDC9	SPAG9	FEZF1	SLC12A2	ANKRD63	OPRD1
CRCT1	IL2	ZNF467	FGF20	SLC12A3	ANKRD65	EIF6
ZNF626	SLC37A2	ZNF23	FLT1	SLC12A6	ANKRD66	BAX
ZNF556	PCNA	HSPA5	FLT3	SLC22A5	ANKRD7	CDK6
HSPA8	ZSCAN21	ID1	FLT4	SLC35A1	ANKRD9	CDKN2A
ZNF541	GALE	ASB8	FMO3	SLC36A2	ANKS1A	EEF1E1
ANKS4B	IDI1	HTR3D	FN1	SLC38A8	ANKS1B	AHSA1
POR	EDNRA	ZNF197	FOS	SLC5A5	ANKS3	ODC1
SNAI1	ZFP1	ZNF860	FXYD2	SLC6A2	ANKS4B	BAD
SP7	CLEC1B	MMP13	FYN	SLC6A3	APCS	HIF1A

NDUFS4	AMHR2	NDOR1	G6PC	SLC6A4	APEH	IGF1R
ZNF785	CHODL	ZNF131	GABBR1	SLC7A11	APOH	RUNX1T1
SLC32A1	CCDC167	ZNF676	GABRA1	SLC7A14	ARGLU1	HERC5
RAC2	SNAPC3	ZNF160	GAL	SLC7A7	ARHGEF7	CDK1
ZBTB44	SREBF1	ANKDD1B	GALC	SLCO2A1	ARSD	ACACA
MPHOSPH8	ZNF382	CKS1B	GAPDH	SMAD2	ARSF	CCND2
ZNF776	FAM129A	CHRM4	GATA1	SMAD3	ARSG	SERPINE1
FMO5	CHRM5	UBA2	GATA2	SMAD9	ARSJ	IKBKG
CDK4	ZNF276	ANKRD30A	GATM	SMC1A	ARSK	CYP19A1
CXCR4	ZNF701	ZNF470	GDF1	SNCA	ARSL	PSMD3
KRT15	UNC119	ZFP14	GFAP	SNCAIP	ARTN	CYCS
RB1	LRRFIP1	ZDHHC13	GFI1	SOD1	ASB1	CFLAR
ZNF846	MAT2A	BCL2A1	GFI1B	SP110	ASB11	AAGAB
RAB6A	CLEC6A	ANKRD10	GJA1	SRC	ASB12	INS
MSMO1	UPP1	TRPM5	GLIS2	SRPX2	ASB13	FCER2
HTR3B	ZNF24	THOC1	GLUD1	ST14	ASB14	IL13
PHF21A	UGT1A4	PIDD	GLUL	STAR	ASB15	ALPI
ZSCAN12	GCH1	ZNF792	GOT1	STAT1	ASB16	PSME3
PLD1	ANKRD24	ZNF610	GRIA1	STAT2	ASB18	G6PC
ANKRD37	NPAS3	COL10A1	GRIA2	STAT3	ASB2	APC
PPP1R27	HSPA4L	SNCAIP	GRIK2	STAT5B	ASB4	TRPM2
ZNF121	ACTRT2	CKMT2	GRIN2A	SYK	ASB5	AKR1C3
MAF	ZBTB45	CD44	GSK3B	TACR1	ASB6	SLC5A5
ANKRD55	ZFP91-CNTF	ARGLU1	GSTM1	TARDBP	ASB7	FXYD2
ZNF766	CHRNA6	ZNF765	GSTP1	TEK	ASB8	ALG5
ASB15	HP1BP3	GPX7	HABP2	TERT	ASB9	soxA
ZNF66	GLI2	ZNF507	HACE1	TF	ASH1L	cumD
BMI1	ZNF180	ZNF671	HBB	TFRC	ASPG	GATM
ESPNL	HLA-B	CHRM2	HCRT	TGFA	ASZ1	FKBP1A
ZFP64	ZNF763	ZNF655	HEXB	TGFB1	ATF3	SRC
ZNF217	REG3G	APP	HGF	TGIF1	ATF4	IGHG2
TH	ZNF410	ZNF578	HIC1	TH	ATG5	KDR
EHMT1	NKPD1	INSIG1	HIF1A	TIMP3	ATHS	BACE1
PEG3	ZNF497	ZNF491	HINT1	TLR4	ATRNL1	TUBB1
VRK2	ANKRD18A	ZNF92	HLA-A	TMPRSS15	AZGP1	NR3C1
ZBTB12	ZNF432	HSPA12A	HLA-B	TMPRSS6	AZU1	MMP13
ANKRD36C	ZNF600	PRDM15	HLA-DQA1	TNF	BACE1	MMP8
GDF1	IL15	ZNF238	HLA-DRB1	TNFRSF1A	BAMBI	SP1
PSMD10	IL1B	ZBTB22	HMGCR	TNFSF11	BANP	ENPP7
HDDC2	CLEC12B	ZDHHC17	HMOX1	TOP1	BCL11B	RXRG
ZSCAN25	GPR50	ZNF576	BCL6B	KLK14	SLC44A2	
BCL6B	ACE	RAI14	BCLAF1	KLK15	SLC44A3	
CDK13	ZNF419	CDKN2B	BDKRB1	KLK2	SLC44A5	
POU2AF1	NACC1	KLRF2	BIRC8	KLK5	SLC5A12	

ANKRD30BL	ZNF408	UQCRFS1	BLCAP	KLK6	SLC5A6
SEC61G	CA13	ANKRD7	BNIP2	KLK7	SLC5A8
MST1	ZIC4	ZNF322	BNIP3	KLK8	SLC7A13
HKR1	CLMP	CA7	BNIP3L	KLK9	SLC7A3
GUCA1A	ZNF101	MECOM	BSPH1	KMT2B	SLC7A6
ZNF597	CHRNB2	CLEC12A	BTBD11	KMT2C	SLC8B1
ZNF484	ANKRD63	CKS2	BTF3	KMT2E	SLC9A3R2
UBE3A	ZNF827	ZBTB46	C1RL	KMT5A	SMYD1
SNCA	TFRC	PLAGL1	CA10	KRBOX1	SMYD4
ACTL6B	ZNF445	ZNF284	CA11	KRBOX4	SMYD5
ZNF705A	TNFRSF1A	HTR7	CA13	KRT15	SNAPC3
ACTRT3	ZNF793	SLC38A4	CA14	KRT80	SNUPN
ZNF705G	TNKS	ZNF460	CA3	LAMA5	SOWAHC
CYP26A1	CGA	KLRG1	CA5B	LAMB4	SP140
GPX8	ZNF579	ZNF446	CA6	LAMC1	SP140L
ZFP3	ZNF208	SFTPA1	CA7	LCE1B	SP2
E2F1	FGF20	ZNF558	CALCB	LCTL	SP3
ZNF670	ZBTB6	ZNF565	CAPZA2	LGALS12	SP4
SLCO2A1	CYP2A13	ZNF350	CARTPT	LGALS13	SP5
ZNF236	STAT2	ZNF423	CASKIN1	LGALS14	SP6
CCR5	CA3	ZNF25	CASKIN2	LGALS16	SP8
ENTPD3	BAD	DNMT1	CASP4	LGALS4	SP9
ABTB1	ZNF274	DNASE2	CBR3	LGALS9B	SPOCK1
SH2D2A	TRIP11	ANKRA2	CBR4	LGALS9C	SPRR2D
IRAK3	COL4A2	SLC38A10	CCDC159	LGALSL	SS18L2
UBE2I	ZNF214	PNMT	CCDC167	LPA	ST20
ZNF555	ASZ1	ASB1	CD1B	LRIT1	STAC
COL5A3	CAPZA2	CYP2C18	CD2	LTF	STEAP1
ZNF70	ZNF571	ACTR3B	CD24	LY86	STK25
IKZF3	ZNF808	SCAF11	CD276	M6PR	STXBP3
ZNF425	ZNF677	ANKRD1	CD46	MAN1A2	SUMO4
ZSCAN10	EPHA3	NANOG	CELA1	MAN1C1	SUV39H1
POU2F1	ZNF628	ZNF708	CELA2A	MAN2A2	SYNPO
ZNF134	HLA-C	PRDM14	CELA2B	MAN2B2	TAF7
ZFP91	ZNF513	ANKRD53	CELA3B	MAP3K20	TAGLN2
TREM1	ZNF592	ZNF3	CFL1	MAZ	TANC1
ZNF844	CASP3	ZNF311	CGA	MED24	TANC2
KLF5	YY1	GLIPR1	CGREF1	MED28	TCF20
ZNF76	ZBTB4	GOLGA4	CHDH	MEGF9	TCFL5
ZNF680	ANKRD20A2	TRA2A	CHGA	MELTF	TERF2
COL6A5	ZNF721	DFFB	CHKA	MEP1B	TFDP1
ZNF75A	CYP4X1	ZNF716	CHKB-CPT1B	MMP15	TGFBR3
MMP2	ZEB1	ZNF391	CHRFAM7A	MMP16	THOC1
HEY1	MYD88	LAMB1	CHRNA10	MMP17	TIMM17A

HIVEP1	FEZF2	ZXDC	CHRNA3	MMP23A	TINAGL1
CA10	IFNAR1	ZBTB33	CHRNA5	MMP25	TIPARP
DVL2	ZNF720	ACTA1	CHRNB3	MMP26	TM7SF2
HTR3A	JUNB	ZNF260	CHRNB4	MPHOSPH8	TMEM41B
ZFP37	PTBP2	CCNB1	CKS1B	MST1R	TMPRSS11A
ZBTB47	ZNF93	ZNF616	CLC	MTA1	TMPRSS11B
ZNF551	PPARG	CYP2U1	CLCC1	MTA3	TMPRSS11D
ZNF649	NR5A1	ZNF611	CLCN3	MTIF2	TMPRSS11E
ZNF540	BAX	ZNF646	CLEC2B	MUC4	TMPRSS11F
ZNF483	GPX3	CA2	CNR1	MYNN	TMPRSS12
KRT85	MMP9	SMAD9	COL16A1	MZF1	TMPRSS2
ITGAL	ZNF594	ZNF75D	COL20A1	N4BP2	TMPRSS3
ASB13	C4BPA	ZNF219	COL22A1	NANOG	TMPRSS4
FOSB	ASB16	ZNF775	COL23A1	NAT10	TMPRSS5
ZNF347	COL9A3	GLIS1	COL24A1	NCOA2	TMPRSS7
ADAM19	ZNF140	ANKFY1	COL5A3	NDOR1	TMPRSS9
ZBTB17	ZNF581	ZFR	COL6A5	NDUFA5	TNFRSF25
ACTA2	ZNF85	CR1L	COL6A6	NECTIN2	TNFRSF6B
ILK	ZNF862	SERPINH1	CORT	NECTIN3	TNFSF14
ZNF257	ALAD	ZNF251	COX5A	NEFM	TNKS
ZNF100	COL27A1	ZNF516	CRCT1	NEO1	TNKS2
POTEB	ZNF148	ZNF546	CRELD2	NFKBIB	TOB1
ZNF599	ZNF114	ZNF280C	CSF1	NFKBID	TONSL
SUPT4H1	ZNF469	ZNF625	CSF2RA	NFKBIE	TOPBP1
CLEC2B	ZNF16	CA4	CTCFL	NFKBIZ	TPSAB1
OAS1	PTAFR	SLC39A6	CTRB2	NHLRC4	TPSD1
ZNF506	CYP2R1	SLC36A2	CTRL	NIBAN1	TPSG1
HLA-G	NOTCH3	SP9	CTTNBP2	NKPD1	TRAF3
ZNF273	ZNF287	RUNX1T1	CXADR	NKRF	TRAF4
ANKRD42	PTX3	KRBOX4	CYP4F3	NLGN1	TREM1
TRIB2	RLF	ZNF644	DAZAP1	NLGN2	TRIB1
CDKN2D	ZNF580	SHANK1	DCAF16	NLGN3	TRIB2
FGF1	ZNF652	CYP2C8	DCP2	NLGN4Y	TRIM10
CLEC2L	CA5B	CCND2	DDB1	NOTCH4	TRIM11
ACTL10	ZNF433	ZBTB40	DEDD	NPAS3	TRIM15
TNFSF11	ZNF500	HLA-A	DEFB129	NRARP	TRIM17
NTRK1	ZNF324	ASB2	DFFB	NSD3	TRIM31
NFKBID	MADD	KLF17	DGKD	NTSR1	TRIM34
ZNF787	ZNF514	COL11A2	DNASE1	NUDT11	TRIM4
LPA	CARTPT	ZSCAN5B	DNASE1L2	NUDT14	TRIM40
GABPB1	CHD2	ZNF613	DNASE2	NUDT5	TRIM41
ZNF414	RPS25	REG1A	DPEP1	NUDT7	TRIM43
YY2	ZNF689	ZNF264	DPP4	NUDT8	TRIM48
FOXM1	GFI1B	PKN2	DPP7	OAS1	TRIM49

MIB1	ZNF10	CLEC5A	DVL2	OGT
ZFP62	ANKRD26	ACTL6A	DYNC1LI2	OLFM4
C3AR1	KLF9	REST	E2F4	OLFML1
ANKRD27	COL5A2	CRH	E4F1	OLFML2A
COL17A1	ZNF146	UACA	EFNA2	OLFML2B
ZNF28	CSF2	PRSS1	EFNA3	ORC2
MTRR	ANKRD18B	MMP7	EHMT2	OSR1
ANKRD39	ACTBL2	ZNF816	ELL2	OVCH1
ZBTB49	CCND1	ZNF77	ELSPBP1	P2RX3
SLC7A5	ZNF678	STAT6	EML4	P2RX5
CLEC3A	ZNF398	ZMYND8	EMP1	P2RX6
ZNF813	ZNF518B	ITGA2B	ENPP2	PANK3
DBH	ZNF200	ANKHD1	ENPP3	PAPLN
ZNF192	REG1B	IL1A	ENPP4	PATZ1
ZNF619	IL7	ZBTB2	ENPP5	PCTP
POU5F1	ZNF416	TFPI2	ENPP6	PCYT1B
ZSCAN5A	ZNF732	DHPS	ENTPD3	PDLIM3
MYL6	DDIT4	CTSH	EPHA3	PEG3
CD209	FEM1C	ASB7	EPHB3	PGM1
ASB11	KANK4	HMGCR	ERG	PHF11
NDUFA5	ZNF681	ZNF30	ERMAP	PHF3
CALR	PATZ1	ZNF292	ERN1	PHF7
FAM83F	STK25	ZNF543	ESPNL	PIDD1
PTHLH	KLF16	ZNF233	ETNK1	PIGF
NOTCH2	CD55	UGP2	ETNK2	PIK3C3
CA5A	HSPA1L	XIAP	EVA1C	PIP
FAH	ZNF133	ZBTB26	EVPLL	PKIA
HDAC2	ZNF585B	ZNF583	FAM20B	PLA2G1B
FOS	ANKRD46	PDGFB	FAM83F	PLAGL2
ZNF668	NRARP	ACTL8	FANK1	PLBD1
POMC	FAT2	ZNF281	FAT2	PLBD2
ZNF136	ZNF473	PSMB6	FBN3	PLD1
KLF7	SP1	EFNA2	FBXO2	PLD2
ACTL9	CYP17A1	LTF	FBXO44	PLD4
ZNF250	ZNF300	ZNF845	FBXO6	PLD6
ZBTB8A	DHFR	COL22A1	FCAR	PLXNA2
RBBP6	ACTG2	ZFP106	FCGR3B	PLXNB1
GATA2	ASB9	ZBTB20	FEM1A	PNMA1
HSPA6	CHRNA3	ACTC1	FEM1B	PNN
ZNF135	ZNF552	ANKS6	FEM1C	PNPLA4
ZNF366	ASB12	PLAG1	FEZ1	POC1B-GALNT4
EML4	ZNF429	COL24A1	FEZF2	POSTN
ZNF548	ZBTB34	ZNF572	FIZ1	POTEB
ALOX5	ZNF20	ANKRD35	FLRT2	POTEB2

ZNF397	ZNF789	CGREF1	FMO1	POTEC
ZNF383	ZNF296	ZNF764	FMO2	POTED
YAP1	F2	ZNF224	FMO4	POTEE
TRNP1	MME	GLI1	FNDC1	POTEH
TAOK1	ZNF823	TNF	FOXA1	POTEI
ZNF726	PLAGL2	ZNF490	FPGT-TNNI3K	POTEJ
ZNF226	ZNF439	KRT6B	FSCN1	PPP1R12A
ZBTB38	CHRNB1	ZNF155	FSD2	PPP1R12C
ZNF711	COL6A6	SLC38A6	GABBR2	PPP1R14A
ZNF688	REPIN1	ZNF449	GABPB2	PPP1R16A
ANKRD17	CSMD1	RPL7A	GALNT12	PPP1R16B
REG4	FPGT-TNNI3K	MAFF	GALNT13	PPP1R27
TYW1	ZSCAN22	ZNF354A	GALNT15	PRDM10
CASKIN2	ZNF501	ZNF512	GALNT17	PRDM13
RNASEL	SIVA1	ZNF496	GALNT18	PRDM14
ZNF614	PIK3CA	ZNF672	GALNT4	PRDM15
hCG_1984214	ZNF22	NME3	GALNT5	PRDM6
ZNF283	ZFPM2	ZNF81	GALNT6	PRDM9
ZNF443	PDGFRB	PTPRG	GALNT7	PRDX6
KCNJ8	ZNF41	ZNF526	GALNT8	PRLH
IKZF4	CA6	ZNF530	GALNT9	PROM2
RFXANK	FCER2	ZNF256	GALNTL5	PROX1
CLIC1	CLEC4G	KLF11	GALNTL6	PROZ
C5AR1	BCL11B	CBX3	GAST	PRPF38B
ZNF664	INVS	ZNF394	GDF15	PRSS21
RAF1	JUN	ZNF331	GFRA3	PRSS22
POTEC	VCAN	NOS3	GLB1L	PRSS27
ZNF587	ZFP36	ZNF468	GLB1L2	PRSS3
ZNF132	ZNF714	SPRR2D	GLI4	PRSS33
GPX2	ZNF205	ZNF26	GLIS1	PRSS36
BCL2	PHGDH	ACTR2	GLTP	PRSS37
ZNF653	HSPA1B	ATRX	GNG10	PRSS38
GLI3	KRT80	CHRNA1	GOLGA4	PRSS40A
ZNF175	ZNF746	WNT7B	GPNMB	PRSS42P
MLPH	ZNF341	IGFBP2	GPR50	PRSS45P
IKZF1	ZNF705D	ZBTB7B	GRAMD1B	PRSS46P
SLC38A2	MMP3	CYP2J2	GRIA4	PRSS48
ZBTB41	CD59	AMD1	GRIK1	PRSS50
ZNF841	PRLH	ZNF32	GRIK3	PRSS53
PNPLA4	TJP1	FMO2	GRIK5	PRSS54
ZNF836	ZNF471	ZNF816-ZNF321P	GSDMA	PRSS55
ZNF529	CDKN1A	ZSCAN18	GSPT1	PRSS57
ANKRD23	CDK6	ALDH1A1	GSPT2	PRSS58
ZNF225	ACTG1	ZNF492	GTF2H1	PRSS8

ASCL1	KLF2	ZNF574	GTF3A	PRTG
CHRNA5	ZNF263	ZNF169	GTF3C4	PSMD10
NR3C1	GPX4	ZNF235	GZF1	PTBP2
ANK3	UFM1	E4F1	GZMA	PTPRA
ZNF577	POTED	HSPA4	GZMH	PTPRZ1
TP53	ANKRD29	ZXDA	GZMK	PTX3
FGF9	GSPT1	ZBTB10	GZMM	QPCT
GSPT2	KCNA5	ZSCAN30	HAVCR1	QPCTL
ZNF800	VWA1	HMGA1	HDAC2	RAB6A
STEAP1	PPP1R12C	ZNF174	HDAC7	RAB9A
SCAND1	ZFP90	NKRF	HDDC2	RAD9A
ZNF404	CHRNG	ZNF586	HGFAC	RAET1E
RREB1	ZNF367	TONSL	HIC2	RAI14
CLEC11A	SERINC3	CSMD3	HLA-C	RAN
CLEC9A	ZNF582	ANKS1B	HLA-DMA	RBAK
ZKSCAN1	ZFP82	ATMIN	HLA-F	RBFOX2
ZNF528	ZNF696	ZNF852	HLA-G	REL
ZNF788	HIC2	PIGF	HNRNPH2	REPIN1
COL1A1	SLC36A1	NIN	HOXB9	REV1
ZNF83	HSPA1A	UBE2C	HP1BP3	RFPL1
ZNF184	ZNF853	OPRK1	HPGDS	RFPL2
POTEB2	ZXDB	CTSK	HPN	RFPL3
ZNF362	ZNF384	SLC36A3	HPR	RFPL4A
CA12	NFYB	CASP9	HPX	RFPL4AL1
ZNF112	GRAMD1B	ZNF493	HSP90AA1	RFPL4B
ZNF436	ZNF333	ACTR1B	HSP90B1	RIPK1
ZIK1	PRPF38B	ZNF596	HTR3B	RIPK2
HCRT	EIF2AK3	BRCA1	HTR3C	RNF112
ZNF79	ZFP69	ZNF234	HTR3D	RNF186
CYP3A4	GLI4	CD207	HTR3E	RNF39
ZNF479	CSMD2	KLF3	HTR7	RPS25
FN1	ZNF354B	HDAC1	HUS1	S100A10
ZNF799	CCNE1	CORT	ICAM5	S100A14
ANKRD54	KIF21B	WISP2	IFI44	S100A2
PLAT	MZF1	COL11A1	IFI44L	S100A4
FAT1	RBM25	IVL	IFNAR1	SAMD7
FIZ1	KLK5	ZNF317	IFNL1	SCAND1
LGALS1	ZNF665	SOS1	IFNLR1	SCGB2B2
RUNX2	ZNF117	MED24	IGDCC4	SDK1
TFDP1	COL9A1	PRKCA	IGKV3D-7	SEC11A
SLC36A4	CYP2C19	PRKCZ	IGSF9B	SEC11C
MT1E	PRG3	ZIC5	IKZF2	SEC61G
HIVEP2	KLF8	CYP2W1	IKZF3	SEMA4C
ZNF564	ZNF329	P450-CYP21B	IKZF4	SEMA4F

POTEE	CHRM1	ERG	IKZF5	SEMA5B
EHMT2	PLD2	MMP11	IL22	SEMA6C
ZBTB39	CA11	EZH1	IL22RA1	SEMA6D
ZNF865	CCK	MMP19	IL34	SEMA7A
SLC9A3R2	ZNF319	MT1A	IL3RA	SERINC3
CHRNA4	ZBTB5	LMNA	IL7	SETD1A
TANC1	ZNF629	HPGDS	IL9	SETD1B
OLFM4	CYP1A2	MMP17	ILK	SETDB1
ST20	ANKRD2	KMT5A	IMMP1L	SETDB2
CTCF	STIP1	MMP21	IMMP2L	SETMAR
POTEI	CHRFAM7A	GSR	ING3	SH2D2A
GLIS2	ARC	GADD45A	INS-IGF2	SH3KBP1
ZNF654	PON1	ELSPBP1	IRAK1	SH3RF3
ZNF524	PPP1R12A	MGMT	IRAK3	SHANK1
STAT3	ZNF607	MMP16	JAML	SHANK2
UNC5B	HLTF	PRG4	KANK3	SIVA1
ZNF724	ZFP161	SETD1B	KANK4	SLC12A8
ANKS3	PHF3	GSTA3	KDM7A	SLC12A9
OGT	ZCCHC11	SETBP1	KIDINS220	SLC25A48
RGS1	ZNF573	NFE2L3	KIF21B	SLC32A1
ZNF625-ZNF20	ZNF669	DNMT3B	KIRREL1	SLC36A3
ZNF12	ZNF525	SMYD2	KIRREL2	SLC36A4
C7orf50	S100A14	SMYD1	KLB	SLC37A2
SLC25A48	GPX1	TXN	KLF15	SLC38A1
FASLG	CDKN2C	MMP10	KLF16	SLC38A10
ZCCHC9	ZFP42	REL	KLF17	SLC38A11
ANK1	SP3	HDAC6	KLF8	SLC38A6
HMOX1	FOLR1	MLL5	KLK1	SLC38A7
ZNF211	ZBED9	GSTM1	KLK11	SLC38A9
ZFP57	ZNF517	SETD2	KLK12	SLC39A6
TINAGL1	CD69	MLL2	KLK13	SLC44A1
SETD1A	HTR2C	TGFA	ZNF263	ZNF749
MMP8	TBXA2R	IL23A	ZNF264	ZNF75A
SETDB2	ACOT4	RNF112	ZNF268	ZNF75D
PHF7	F2RL3	LGALS12	ZNF273	ZNF76
SMYD5	XCR1	RFPL4A	ZNF276	ZNF763
KLK3	PTGER1	TRIM69	ZNF28	ZNF764
NDRG1	NPSR1	ADORA2B	ZNF280A	ZNF765
ERCC5	PRSS55	ENPP2	ZNF280C	ZNF766
MT2A	C1RL	SEMA6D	ZNF280D	ZNF77
AKR1B10	OVCH1	AMIGO2	ZNF281	ZNF770
MMP23A	BAAT	SEMA7A	ZNF283	ZNF771
NFE2L1	ELANE	RDH10	ZNF284	ZNF772
UGT1A1	GNA15	SFRP1	ZNF286A	ZNF773

CBR3	GRP	SEMA3F	ZNF286B	ZNF774
STAT5A	KLK1	TRIM49B	ZNF287	ZNF775
GSTA2	GNRH2	SFRP5	ZNF292	ZNF776
KEAP1	F7	TYRO3	ZNF296	ZNF777
GCLM	GPR65	PTGER4	ZNF3	ZNF778
GCLC	MTRNR2L12	CPO	ZNF30	ZNF780A
PHF11	KNG1	HLA-DQB1	ZNF300	ZNF780B
GSTP1	OXT	TRIM10	ZNF302	ZNF781
AKR1C1	QRFPR	CSF1	ZNF304	ZNF782
MMP24	NPS	PASK	ZNF311	ZNF783
SOX9	PMCH	FLT4	ZNF317	ZNF784
WHSC1L1	PRSS54	IL2RG	ZNF319	ZNF785
TCF20	GCGR	LGALS8	ZNF32	ZNF786
MMP20	GPR132	CCR4	ZNF320	ZNF787
SUV39H1	GZMA	FUCA2	ZNF322	ZNF788P
AR	PRSS27	CSK	ZNF324	ZNF789
NFE2L2	P2RY2	NTN4	ZNF324B	ZNF790
MMP26	PRSS46	EFNA4	ZNF329	ZNF791
VTN	GPR68	LAMA3	ZNF333	ZNF792
MMP14	EDN3	AMPD1	ZNF334	ZNF793
SETDB1	C1S	NUDT14	ZNF33A	ZNF799
EZH2	GPR17	HSD11B1L	ZNF34	ZNF80
HPX	TMPRSS15	TRIM61	ZNF341	ZNF800
PTGES	ACSL5	ABCB9	ZNF343	ZNF805
CES1	PAEP	PVRL2	ZNF345	ZNF808
SETD7	ACSBG1	GALR1	ZNF347	ZNF813
JAK2	GLTP	NPR3	ZNF35	ZNF814
MMP25	ACSL1	GPR52	ZNF350	ZNF816
MLL	ALDH9A1	TRIM58	ZNF354A	ZNF816-ZNF321P
ABCC2	IAH1	FSD2	ZNF354B	ZNF823
ERCC4	ACSL4	GPR101	ZNF354C	ZNF827
BSPH1	ACACB	PGA3	ZNF362	ZNF829
RELA	MBOAT4	NUDT10	ZNF366	ZNF83
WHSC1	LIPT2	CD1D	ZNF37A	ZNF831
AKR1C3	ACSL3	RDH11	ZNF382	ZNF835
TLR4	PDHA1	IL12A	ZNF383	ZNF836
MMP27	ALDH3A2	GPR83	ZNF384	ZNF837
AKR1C2	ALDH1B1	B4GALT1	ZNF391	ZNF841
SETMAR	PPARA	GPR45	ZNF394	ZNF843
NQO1	RPS6KA3	GRIN3B	ZNF396	ZNF844
MAPK14	TNFRSF10A	TRIM7	ZNF397	ZNF845
MMP15	TRPV3	ICAM5	ZNF398	ZNF846
NQO2	TRPV2	RXFP4	ZNF404	ZNF850
ASH1L	ERBB2	CES4A	ZNF407	ZNF852

PRDX5	CFLAR	TAP1	ZNF410	ZNF853
MAPK8	CASP7	MC5R	ZNF414	ZNF860
SMYD4	WNT5A	NAPSA	ZNF415	ZNF862
TXNRD1	BNIP3	CPA1	ZNF416	ZNF865
SUV39H2	IGF1	CSTA	ZNF417	ZNF875
GSTA1	BIRC8	LGALS9	ZNF418	ZNF878
NSD1	ERN1	FNDC1	ZNF419	ZNF879
CBR4	FABP4	NRXN3	ZNF420	ZNF880
GSTA4	TIMP3	ROR1	ZNF425	ZNF90
AZIN1	ACOX1	DHRS2	ZNF426	ZNF92
AKR1D1	PRDX6	TRIM21	ZNF429	ZNF93
ADC	TEK	TRIB3	ZNF43	ZNF98
ODC1	SCP2	CPB2	ZNF431	ZNF99
TYR	TPI1	TLR8	ZNF432	ZSCAN1
PRSS8	BAG3	PCSK5	ZNF433	ZSCAN10
GCG	COL18A1	LMAN2	ZNF436	ZSCAN12
EDNRB	HRAS	PRLHR	ZNF439	ZSCAN16
TMPRSS7	GAL	PIGN	ZNF440	ZSCAN18
PRSS3	COX5A	PTGDR	ZNF441	ZSCAN2
LTB4R2	HSP90B1	LGALS9C	ZNF444	ZSCAN21
LPAR6	TRAF3	INSR	ZNF445	ZSCAN22
F2RL1	CASP4	EFNB3	ZNF446	ZSCAN23
TACR1	DDIT3	ABP1	ZNF449	ZSCAN25
EDN2	CDK2	AMPD3	ZNF454	ZSCAN29
HCRTR2	CASP8	HSD3B7	ZNF460	ZSCAN30
HRH1	IGFBP1	EGF	ZNF461	ZSCAN31
TACR3	PRKDC	NUDT5	ZNF462	ZSCAN32
TMPRSS11F	MAPK9	FGA	ZNF467	ZSCAN4
TMPRSS11D	NPC1	ERMAP	ZNF468	ZSCAN5A
KLK2	MAP2K1	HEXB	ZNF470	ZSCAN5B
KLK12	ABL1	MPO	ZNF471	ZSCAN5C
CTRL	PCTP	SP140	ZNF473	ZSCAN9
AZU1	S100A4	RFPL4B	ZNF479	ZXDA
LTB4R	BANP	NHLRC4	ZNF48	ZXDB
LPAR1	FABP5	SEMA5B	ZNF480	ZXDC
UTS2	TRPV4	TRIM48	ZNF483	TRIM49B
XCL2	TNFRSF25	OS9	ZNF484	TRIM49C
NPFFR2	PGM1	PCSK7	ZNF485	TRIM51
KLK15	CBS	GPR12	ZNF486	TRIM58
TMPRSS11A	CALCB	FZD4	ZNF490	TRIM6-TRIM34
HTR2B	SUMO4	FCER1A	ZNF491	TRIM60
CCKAR	TNFSF10	TRIM11	ZNF493	TRIM61
NMU	FOXA1	RIPK3	ZNF496	TRIM64B
GRPR	TRPV6	USH2A	ZNF497	TRIM64C

KLK9	CHEK2	DHRS1	ZNF500	TRIM66
PRSS57	BID	IL28RA	ZNF501	TRIM68
PRSS48	CALCA	TRIM72	ZNF502	TRIM69
CELA2A	FASN	DNASE1L1	ZNF507	TRIM7
NTS	PTGS1	GALR2	ZNF510	TRIM72
PROZ	JAK1	CTSA	ZNF512	TRIM73
ADRA1A	TRAF4	C2orf81	ZNF514	TRIM74
FFAR1	TRPV1	TRIM49C	ZNF516	TRIML1
AGTR1	GDF15	PIGG	ZNF517	TRIML2
PRSS42	TIMP1	EPHA4	ZNF518B	TRNP1
CTRB1	MAP3K7	GPR84	ZNF519	TRPV2
ABHD12	OCLN	TGFBR2	ZNF521	TRPV5
CORIN	GLUD1	GPR88	ZNF524	TRPV6
CFD	BNIP2	EPHA1	ZNF525	TUT4
KLK8	NOX3	C5	ZNF526	TYW1
MASP1	DFFA	SH3RF3	ZNF527	UACA
TMPRSS9	PPARD	SIGLEC7	ZNF528	UBA2
QRFP	IL12B	IL21	ZNF529	UBE2I
PRTN3	TGFB1	ADRA2C	ZNF530	UFM1
ABHD17B	RIPK2	KIRREL3	ZNF532	UNC5B
F11	MCL1	FSHR	ZNF534	UQCRFS1
PRSS58	TNFRSF10B	GPR37	ZNF536	VEGFD
F12	STAT1	BRS3	ZNF540	VRK2
LPAR2	ALB	CPA3	ZNF541	VSIG4
UTS2B	BAK1	MAP3K21	ZNF543	WIZ
GZMM	ATG5	GLA	ZNF546	WNT7B
TRHR	IGF1R	SELL	ZNF547	XPO5
HP	CPT1A	FZD7	ZNF548	YES1
GNRHR	MAP2	HTR6	ZNF549	YY2
HCRTR1	ATM	FBXO6	ZNF550	ZBED9
KLK13	PARP1	M6PR	ZNF551	ZBTB12
KLK7	TM7SF2	AVPR1B	ZNF552	ZBTB14
MCHR2	RXRA	CASQ2	ZNF554	ZBTB2
GRM1	ANGPT2	FOLH1	ZNF555	ZBTB22
ACOT6	GFAP	AMY2A	ZNF556	ZBTB26
PRSS21	BCLAF1	TLR3	ZNF557	ZBTB3
CTNNBL1	E2F4	LYN	ZNF558	ZBTB32
CTRB2	SLC12A2	ACE2	ZNF559	ZBTB33
PROK1	RXRB	TRIM64B	ZNF559-ZNF177	ZBTB34
NMUR2	PKM2	ACVR2B	ZNF560	ZBTB37
PTGFR	ABCB1	MAN1A1	ZNF563	ZBTB39
GZMH	EGFR	FGFR4	ZNF564	ZBTB4
HGFAC	BNIP3L	C8A	ZNF565	ZBTB40

TISP43	GAPDH	ABCC6	ZNF566	ZBTB41
SAA1	CFL1	HTR4	ZNF567	ZBTB43
MLNR	ATF4	CNR2	ZNF568	ZBTB44
NPFF	APAF1	HTR1B	ZNF569	ZBTB45
AKR1B1	SIRT1	SAMD7	ZNF57	ZBTB46
PRSS12	ENOX2	EFNB2	ZNF570	ZBTB47
ACOT1	PRKCD	C4B	ZNF571	ZBTB48
CELA2B	TRPV5	CDC42BPB	ZNF572	ZBTB49
CMA1	ANGPT1	RFPL2	ZNF574	ZBTB5
C2	CHKB	BACE2	ZNF575	ZBTB6
TPSD1	BDNF	HSD17B8	ZNF576	ZBTB7B
TMPRSS5	SLC5A5	IRAK2	ZNF577	ZBTB8A
ABHD13	ADH6	CSF2RA	ZNF578	ZBTB9
CELA3A	BCHE	PVR	ZNF579	ZCCHC9
F2R	LYZ	PCSK1	ZNF580	ZDHHC13
F9	ADH7	OPN1SW	ZNF581	ZDHHC17
ABHD17C	SLC44A2	SFRP2	ZNF582	ZFAT
ACR	SLC5A8	TRIM15	ZNF583	ZFP1
GNG2	PPARGC1A	NPY5R	ZNF584	ZFP14
PRSS53	PHOSPHO1	RFPL1	ZNF585A	ZFP2
GZMB	SLC44A5	TRIB1	ZNF585B	ZFP28
PRSS36	ADH5	KIRREL2	ZNF586	ZFP3
KLK14	PLD4	LINGO1	ZNF587	ZFP30
KISS1R	PLD3	OPN5	ZNF587B	ZFP37
ABHD12B	ETNK1	GRIN1	ZNF589	ZFP41
FFAR3	SLC22A1	SEMA4F	ZNF594	ZFP42
PRSS50	PTDSS1	DNASE1	ZNF596	ZFP62
HPN	SLC44A1	RDH12	ZNF597	ZFP64
PRSS37	PLD6	CASQ1	ZNF599	ZFP69
OXTR	SLC5A6	LPO	ZNF600	ZFP69B
GNA11	SLC5A7	RNF135	ZNF605	ZFP82
TMPRSS13	GPCPD1	CD2	ZNF606	ZFP90
NMS	PCYT1B	RFPL3	ZNF607	ZFP91
TPSAB1	GRIN2A	MC4R	ZNF610	ZFP91-CNTF
KLKB1	CHDH	SEMA3G	ZNF611	ZFP92
CFI	ADH1A	IGF2	ZNF613	ZFPM1
KISS1	REN	HRH4	ZNF614	ZFR
CELA1	CHKB-CPT1B	CPA6	ZNF615	ZIC4
GNAQ	PCYT1A	GPR3	ZNF616	ZIC5
ST14	SLC22A2	CFTR	ZNF619	ZIK1
GNA14	SLC44A3	GPR63	ZNF620	ZIM3
NMUR1	SLC5A12	LGALS13	ZNF621	ZKSCAN2
ANXA1	BHMT	GPR19	ZNF624	ZKSCAN3
TPSG1	ACHE	AGTPBP1	ZNF625	ZKSCAN4

KLK6	ADH1B	ENPP5	ZNF625-ZNF20	ZKSCAN5
MLN	CHKA	IGLL5	ZNF626	ZKSCAN7
GRB2	ETNK2	SP110	ZNF628	ZKSCAN8
PRSS33	ADH4	MELK	ZNF629	ZMYND8
NMBR	CD1B	DECR2	ZNF630	ZNF100
FFAR2	SLC7A6	SEMA3C	ZNF641	ZNF101
PROK2	SLC7A14	HUNK	ZNF646	ZNF106
AVPR1A	SLC12A4	TESK1	ZNF648	ZNF107
CYSLTR2	SLC7A1	AGA	ZNF649	ZNF112
ABHD17A	SLC7A3	ARTN	ZNF652	ZNF114
PROKR2	SLC12A7	ADRB3	ZNF653	ZNF121
TMPRSS11B	SLC7A8	IRAK4	ZNF655	ZNF124
NMB	SLC7A9	GPR151	ZNF66	ZNF132
GRM5	SLC7A4	RTN4R	ZNF660	ZNF133
KLK10	SLC12A5	HCK	ZNF662	ZNF134
ADRA1D	SLC12A6	GPR148	ZNF664	ZNF135
TAC3	SLC7A7	ZAP70	ZNF667	ZNF136
OPN4	SLC12A3	ITK	ZNF668	ZNF138
CFB	SLC12A1	MAN1A2	ZNF669	ZNF14
GNRH1	SLC12A9	GPR139	ZNF670	ZNF140
PRSS38	SLC12A8	ABCB11	ZNF671	ZNF142
GHSR	SLC7A10	CD1A	ZNF672	ZNF143
TMPRSS2	SLC7A13	MAN1B1	ZNF674	ZNF146
ADRA1B	SLC7A2	TRIM6-TRIM34	ZNF675	ZNF154
P2RY6	PLA2G1B	RNF39	ZNF676	ZNF155
TMPRSS6	PGD	GLB1L	ZNF677	ZNF157
MASP2	DECR1	PGC	ZNF678	ZNF16
UTS2R	CYCS	CES2	ZNF679	ZNF160
KLK11	CXCL3	FGFR2	ZNF680	ZNF169
BDKRB1	CXCL1	TAAR8	ZNF681	ZNF17
TMPRSS11E	G6PD	FAM20B	ZNF682	ZNF174
CTRC	ACOT7	ABCC9	ZNF684	ZNF175
HPR	HNF4A	FZD6	ZNF688	ZNF18
NTSR2	APOB	GPR61	ZNF689	ZNF181
F2RL2	OLAH	SP140L	ZNF69	ZNF184
GAST	CYP4A11	ST6GAL1	ZNF695	ZNF189
TMPRSS4	C8G	MEP1B	ZNF696	ZNF19
GPR4	VLDLR	ENPP4	ZNF697	ZNF197
PRSS22	CYP4Z1	GALNTL6	ZNF699	ZNF20
GZMK	LY96	ACVR2A	ZNF70	ZNF205
FPR2	GIP	TNFSF14	ZNF700	ZNF208
CCKBR	PLA2G2A	TRIM26	ZNF701	ZNF211
CTSG	TLR2	LGALS2	ZNF705A	ZNF213
P2RY10	RXFP3	ERBB3	ZNF705B	ZNF214

TRH	C1QTNF5	MBTPS1	ZNF705D	ZNF215
TAC1	IL6R	GALNT3	ZNF705G	ZNF22
PRSS45	ABCC3	HEPHL1	ZNF707	ZNF221
P2RY1	SDK1	ITGB2	ZNF708	ZNF222
CELA3B	IGF2R	CBR1	ZNF709	ZNF223
CYSLTR1	HLA-DMA	KIT	ZNF71	ZNF224
F10	TRIM4	CD1E	ZNF710	ZNF225
ACOT2	RSPO3	GALNT10	ZNF713	ZNF227
CASR	DCC	SEMA4A	ZNF714	ZNF23
LPAR5	NTNG1	EPHB4	ZNF716	ZNF230
XCL1	TSSK1B	PLBD1	ZNF720	ZNF233
KLK4	SEMA4D	LGALS9B	ZNF721	ZNF234
OXSM	GPR119	IRAK1	ZNF724	ZNF235
MCHR1	BDH2	TRIM60	ZNF726	ZNF236
NPFFR1	B2M	CSF2RB	ZNF728	ZNF24
TACR2	MERTK	HSD3B2	ZNF729	ZNF248
TMPRSS12	SSTR5	DCP2	ZNF732	ZNF25
PROC	TMIGD3	GPR22	ZNF736	ZNF250
EDN1	CD180	TG	ZNF737	ZNF251
PROKR1	TRIM68	IGDCC3	ZNF738	ZNF253
GPRC6A	TNFRSF6B	CTSL	ZNF740	ZNF254
TMPRSS3	MAP3K10	RRH	ZNF746	ZNF256
LPAR3	ABCC5	RSPO4	ZNF747	ZNF257
TRIM66	GPR135	GNAI2		
TRIM40	MATK	S1PR5		
NPBWR1	LGALSL	CCR8		
ADRA2A	POC1B-GALNT4	TAS2R5		
FCGR3B	C4A	HEBP1		
SLC3A2	JAK3	OXGR1		
EFNA3	MAP3K20	SST		
HLA-DQA1	FZD5	GPR55		
GPR173	GPR37L1	HCAR2		
EPHB6	AVPR2	NPW		
AMPD2	GBA	TAS2R14		
TSTA3	MEFV	TAS2R3		
FGR	GALNT4	CCL21		
AMY1A	FZD2	GNAT3		
NSDHL	PCSK4	GRM7		
KDR	ENPP3	TAS2R30		
AZGP1	IL2RA	CCL25		
GLB1L2	HSD17B1	TAS2R42		
CXADR	HTR1E	TAS2R9		
LGALS3	ENPP1	TAS2R46		

LGALS7	TRIM51	P2RY13
LAMA1	HYAL1	MTNR1A
LY86	GFRA3	TAS2R60
LAMB4	GRIK2	TAS2R7
NUDT7	SEMA4G	GRM4
DRD5	MAP3K9	TAS2R16
GPR26	CCR3	GRM2
ERAP1	HSD17B11	CXCL11
IL29	STK11	CXCR1
P2RX1	GPR150	P2RY12
A2M	SFRP4	TAS2R38
ITGA4	LGALS16	CCR7
FCN2	VSIG4	GRM6
IGKV3D-7	AGBL5	TAS2R8
FBXO2	RELN	TAS2R4
CCR1	FGFR3	GNAI1
APCS	EPHA2	PDYN
EPHB1	FZD1	TAS2R41
ADORA1	P2RX3	TAS2R10
SEMA4B	GPR85	TAS2R39
MST1R	PRTG	CXCL10
STYK1	ABCC12	PPBP
ADAL	CHI3L1	TAS2R43
IFNG	BACE1	AFP
ABCB8	FCGR2B	ARSK
NIM1K	SSTR2	NR0B2
MC2R	DNASE1L2	SRSF1
CLC	SORT1	ID3
IL6ST	RNASE1	XPO6
ABCB4	AOC3	NFYA
GRIK1	RIPK1	XPO1
MET	IL34	ESR1
PTGER2	APLNR	DUSP1
PGA4	SRMS	EIF2C2
HSD17B13	FZD9	TUFT1
ABCC11	TRIM73	CHST3
CCR9	PDGFRA	GTF3C4
OPN3	ABCC10	SS18L2
TAAR2	LIMK2	ARSD
SEMA3E	HEPH	FOSL1
GRIK5	GP1BA	HAVCR1
AGBL1	GRIK3	TNFAIP3
GRIA4	ITGA5	ARSJ
HSD11B1	GPR176	BLCAP

KLB	HTR1A	ETS2
TAAR1	CECR1	TIMM17A
P2RX4	DHRS4L2	MRPL49
GALNT12	FCGR2A	ALPL
WBSCR17	GALNT7	CCL2
IL21R	RDH14	RAB9A
AXL	CTLA4	RRAGA
HPGD	AMIGO1	SULT1A1
EPHA7	ABCB5	TNFRSF21
IL22RA1	MAN2A1	PON3
ENPP7	SCARB2	TOB1
NEO1	MC3R	SNUPN
FZD3	TESK2	TCFL5
GALNT1	FSHB	ARSF
GPR78	PCSK2	RAN
TRIM31	LAMB2	HES1
CES3	O3FAR1	DDX3X
BMP7	RGR	BAMBI
EPHA6	HTR5A	KPNA4
OPRD1	INS-IGF2	TGIF1
NUDT1	CTSC	NUF2
GLB1L3	ADA	PIK3CD
MFI2	TYK2	LUC7L3
NUDT8	AMICA1	SIK1
PTGIR	SEMA6A	MT1X
EPHB2	SERPINA3	ARSE
MAN1C1	DHRS7C	PER2
PCSK9	FGFRL1	NDC80
MAP3K11	TRIM74	PTER
PKDCC	LMAN2L	HNRNPH2
LRIT1	FGFR1	PRKACA
ERBB4	PIM3	BHLHE40
RFPL4AL1	HRH2	TARDBP
FUCA1	FRZB	ARSG
RDH8	KL	CALU
OPRL1	HSDL2	MGAT2
PBK	MOS	SRSF5
AGTR2	DHRS7B	TAF7
C6	HLA-DRB1	ELL2
F8	GRIN2D	PNMA1
FCGRT	DRD3	ING3
PTK6	GALNT6	MT1H
FLT1	ABCC4	UGT1A6
GSK3B	GPR161	XPO5

ACPP	CSF1R	UGCG
ABCC8	AGBL3	SULT1A3
OPN1LW	GALNT13	PELI1
INSRR	FGG	VNN1
TRIM41	CUBN	DAZAP1
FAM20A	ITGB7	SULT1A2
ITGAV	ABCB10	PPM1D
C8B	CTSE	TERF2
IGSF9B	SSTR1	LRP5
LCN2	SEMA5A	PNN
CD1C	EFNA5	GPR64
CEL	MANBA	ADGRD1
IL22	FURIN	EVA1C
GALNT8	ITGAX	ADGRG5
ABL2	PLXNA2	ADGRG7
PAPLN	GALNTL4	IMMP2L
PTGER3	TGFBR3	EMR1
CPB1	PNLIPRP1	ADGRF1
GPR27	FGB	OLFML1
FCGR3A	CHIT1	ADGRG3
AMY2B	GPR142	LPHN2
PIP	LGALS4	LPHN1
CLPS	BLK	SEC11C
P2RX6	TRIM34	ELTD1
GPR6	LRRC4C	CD97
SEMA6C	NLGN1	ADGRF2
IDUA	FBXO44	IMMP1L
P2RX5	VTCN1	OLFML3
DHRSX	DHRS4	GPR126
TRIML1	ERN2	OLFML2A
NLGN4X	CHIA	LPHN3
CES5A	CHI3L2	ADGRG4
CXCR7	GALNTL5	EMR2
TRIM64C	IL2RB	SEC11A
TRIML2	MFRP	ADGRF4
MEGF9	SDR42E1	ADGRF5
KIRREL	PVRL3	ADGRD2
ADORA2A	TAAR5	ADGRE3
NLGN2	TRIM49	FBN3
HSD3B1	LCT	ADGRF3
OVGP1	GALNT9	OLFML2B
MINPP1	GALC	ABCG5
CTSD	PML	ABCG8
MC1R	IGDCC4	SLCO1B1

CP	HTR1F
MUSK	GLB1
TAP2	PIM1
NLGN4Y	QPCT
DPP7	DHRS12
DRD4	SDR16C5
TSSK2	RNF186
LIMK1	HSD17B14
TSSK4	ADAMTS13
SSTR4	TEC
GRIN2C	SEMA4C
BMX	SERPIND1
FZD8	PIM2
OPRM1	TRIM43
GPR62	TAAR6
DPP4	EFNB1
GRIA1	NLGN3
BMPR2	CCR2
LAMB3	PECR
DSTYK	NPY2R
LCTL	PPYR1
DRD2	TP73
EPHA10	HUS1
NUDT11	CDK7
AGBL4	OGG1
UXS1	DMC1
NRXN2	GADD45G
GIF	RAD51
CPA4	MAPK12
FIGF	REV1
FAM20C	SESN1
CFH	RAD51B
C3	PMS2
NPY1R	BTG2
LAMA2	N4BP2
MAN2B2	PNKP
RET	SMC1A
RDH13	ERCC1
FZD10	XRCC2
TXK	ERCC2
HEXA	GTF2H2
MAN2A2	MLH3
LGR5	RAD9A
GRIA2	MSH3

QPCTL	MRE11A
EPHA5	NBN
MLKL	PPP1R15A
FCAR	IGHMBP2
NPBWR2	MUTYH
GABRB3	XRCC3
SMO	FEN1
DPP6	MT-CYB
GPR21	CLCN2
PLXNB1	SLC24A6
DHRS13	CLCN5
GSK3A	CLCNKA
CPA2	CLCN7
LAMC1	CLCN4
SEMA3D	CLCN6
LSP1	CLCNKB
ENPP6	CLCN1
ABCC1	P2RY14
GALNT5	TAS2R40
TSSK3	PENK
LGALS14	GRM8
PNLIP	CXCL5
EFNA1	TAS2R13
MAN2B1	P2RY4
PDGFRL	CXCL9
SLC3A1	CXCR5
PTK7	TAS2R19
GALNT15	HCAR3
PLBD2	PTGDR2
PVRL1	CXCL12
OPN1MW2	S1PR3
GALR3	GPR18
NRP1	CCL19
SP100	FPR3
PGA5	CCR10
GPR39	PNOC
FOLR2	CXCL13
SEMA3A	PYY
NRXN1	GRM3
EPHA8	S1PR1
RSPO1	CCL27
HRG	TAS2R20
A2ML1	GPER
ANPEP	CCL28

SI	OXER1
HRH3	TAS2R1
STK40	TAS2R50
RHO	S1PR4
LAMA5	GNAI3
SYK	SUCNR1
NAGA	PPY
GRIN3A	TAS2R31
CXCR6	FPR1
TRIM17	CCL20
SERPINC1	S1PR2
TPP2	NPB
TIE1	MTNR1B
RENBP	CXCL16
GUSB	CXCL6
